# Supplementary material for: Survival and rapid resuscitation permit limited productivity in desert microbial communities
Source: Nat Commun. 2024 Apr 17;15:3056. doi: 10.1038/s41467-024-46920-6 (PMC11519504; doi:10.1038/s41467-024-46920-6)
Supplement: Supplementary file 1 — Supplementary Information [file 41467_2024_46920_MOESM1_ESM.pdf]

Supplementary Material

Survival and rapid resuscitation permit limited productivity in desert microbial communities

Stefanie Imminger, Dimitri V. Meier, Arno Schintlmeister, Anton Legin, Jörg Schnecker, Andreas Richter, Osnat Gillor, Stephanie A. Eichorst, Dagmar Woebken*

*Corresponding Author: Dagmar Woebken

Email address for correspondence: dagmar.woebken@univie.ac.at

This PDF file includes:
Supplementary Notes 1 to 3
Supplementary Figures 1 to 13
Supplementary Tables 1 to 4

Supplementary Note 1

NanoSIMS data analysis

Fundamental aspects about the Inference of biomass generation rates and cellular replication times from heavy water (^2H_2O) labeling experiments

The following theoretical considerations are based on previously published work addressing the inference of cellular assimilation rates from SIP-NanoSIMS data in general (recently reviewed and refined by Polerecky *et al.*[1]) and, more specifically, the application of heavy water (^2H_2O) incubations coupled to NanoSIMS analysis[2,3].

With reference to the designations of the quantities and symbols utilized in the main text (**Methods**), the deuterium content of cells assimilating and incorporating deuterium from deuterated water (F_{incub}) at a certain timepoint (t_{incub}) of the incubation is given by

$$F_{incub} = (a_w F_{D2O} - F_{H2O})(1 - e^{-\bar{\mu} t_{incub}}) + F_{ctrl} \qquad \text{Eq. S1}$$

F_{ctrl} refers to the deuterium content of the cells with natural isotopic abundance (time point zero), F_{H2O} and F_{D2O} designate the deuterium content of isotopically unlabeled water and the water utilized in the SIP incubation. a_w stands for the water hydrogen assimilation constant, which is described more into detail below. $\bar{\mu}$ designates the rate of biomass generation emerging both from cellular growth and regeneration[2,3], *i.e.*

$$\bar{\mu} = \mu_{growth} + \mu_{regeneration} \qquad \text{Eq. S2}$$

More specifically, we refer to the activity in biosynthesis of cellular compounds based on water hydrogen assimilation, presented in inverse units of time (e.g. 1/day). If referred to the hydrogen flux associated with assimilation (e.g. mol hydrogen/day), the instantaneous total cellular hydrogen content needs to be additionally considered, which is challenging to determine for individual cells[1]. Furthermore, we take advantage of the high number of cells that we analyzed (> 210 for each incubation), since Eq. S1 correctly describes the average rate within a community, even under assumption of zero order kinetics, which applies for a constant rate of elemental uptake, irrespective of the cell cycle[1]. As implied by the unit, the inverse of $\bar{\mu}$ yields a generation time. Even in the state of dormancy, continuous degradation and turnover of cellular compounds forces a cell to conduct biosynthesis for maintaining viability. Within a certain time period, which we refer to as regeneration time, the cell conducts a complete turnover of its constituents resulting in doubling of the total biomass although the number of cells remains constant on the community level. This is an important implication for the ecosystem since viable cells, even in the state of dormancy, contribute to the total amount of organic nutrients. E.g. autotrophs participate in replenishment of the organic carbon pool

by carbon fixation and release of cellular compounds. Mathematically, the relationship between regeneration rate ($\mu_{regeneration}$) and regeneration time ($\tau_{regeneration}$) is given by

$$\tau_{regeneration} = 1/\mu_{regeneration} \qquad \text{Eq. S3}$$

If a community is actively growing, the time for biomass doubling is reduced since each replicated cell additionally contributes to biomass generation. Quantitatively, the relationship between the anabolic activity yielding growth (μ_{growth}) and the time for cellular replication ($\tau_{replication}$) is given by

$$\tau_{replication} = ln\ (2)/\mu_{growth} \qquad \text{Eq. S4}$$

It should be noted that even growing cells encounter damage and turnover of compounds, necessitating continuous regeneration. As such, Eq. S2 represents a lower bound for the cellular replication time.

Definition and selection of the water hydrogen assimilation constant (a_w)

For every organism, water represents an essential source of hydrogen for synthesis of specific organic compounds, in particular lipids for membrane formation[2,3,4,5,6,7,8]. Accordingly, deuterated water can be utilized as a universal tracer for monitoring anabolic activity. However, in terms of quantification, there are two constraining factors. The first one is the actual amount of hydrogen originating from water relative to alternative substrates. For autotrophs, it is evident that the entire amount of hydrogen needs to be gained from water and one would expect that the deuterium content of active cells converges towards the deuterium content of the incubation water with ongoing cellular replication. But this is rarely the case due to the second constraining factor, given by kinetic isotope fractionation. Owing to the huge mass difference between the two isotopes, $^2H/^1H$ mass fractionation becomes particularly relevant in reactions including hydrogen atoms. In Eq. S1, both of these factors are combined in the water hydrogen assimilation constant a_w, which reads

$$a_w = x_w \frac{(1-\alpha_{c/w})F_{D2O}}{1-F_{D2O}(1-\alpha_{c/w})} \qquad \text{Eq. S5}$$

where x_w refers to the mole fraction of hydrogen provided by water and $\alpha_{c/w}$ designates the isotope fractionation factor associated with the synthesis of cellular compounds by water hydrogen assimilation. It should be noted that the deuterium content of the incubation water appears in the expression of a_w for obeying mass balance in the net deuterium incorporation reaction (Eq. S1)[3].

In a fundamental study about variations in the hydrogen isotope composition of lipids in four cultured members of the phylum *Proteobacteria*, Zhang *et al.*[8] have shown that water hydrogen assimilation constants can vary over a wide range, depending on the trophic physiology of the organism. The

authors suggested that the observed variation relies on the isotopic composition of NAD(P)H linking catabolism of organic substrates with *de novo* lipid synthesis, which was further investigated and confirmed by Wijker *et al.*[6] for bacterial heterotrophs. Based on the potential range of x_w and $\alpha_{c/w}$ values reported by Zhang *et al* [8] and by considering the deuterium content of the water in our incubations (30 mol%), we calculated upper and lower bounds for $a_w = 0.79$ for obligate chemoautotrophy and $a_w = 0.28$ for obligate heterotrophy.

Sources of bias, definition and selection of the NanoSIMS correction factor (k)

As for any analytical technique, ^2H_2O incubation coupled to NanoSIMS can be subject to bias. In the following, potential sources of systematic error will be discussed.

First, let us rewrite Eq S1 as

$$(F_{incub} - F_{ctrl}) = (a_w F_{D2O} - F_{H2O})(1 - e^{-\bar{\mu} t_{incub.}}) \qquad \text{Eq. S6}$$

which is helpful for our further treatment since the bracketed term on the left now contains the quantities which are experimentally determined.

As already mentioned above, a substantial amount of deuterium taken up from water is incorporated into lipids via *de novo* synthesis. Consequently, if organisms conduct lipid recycling, their metabolic activity is underestimated if inferred solely from water assimilation. The same effect occurs when isotopically labeled metabolites are utilized for cellular growth. Photo-autotrophic Cyanobacteria have been described as being capable of lipid recycling and for managing growth in the dark on account of intracellular metabolites accumulated during (daylight) illumination[9]. This may be the main reason for the constancy of the deuterium content that we observed for the filamentous cyanobacteria between timepoint 12 and 24 hours of the incubation (dark period).

On the contrary, metabolic activity is overestimated by abiotic hydrogen isotope exchange between acidic functional groups in the biomolecules and deuterated water. This effect is, however, reversible upon exposure to isotopically unlabeled water, which is usually part of sample preparation prior to analysis.

Next, let us consider sources of bias introduced through NanoSIMS analysis. We first measured the ^2H content of the cells from the dead control, yielding 0.021 +/-0.006 at% (mean, +/- 1SD). This is slightly above the range of natural variation specified by IUPAC[10]. In principle, such a deviation can result from instrumental mass fraction (IMF) in the NanoSIMS measurement process. If this is the case, the measurement values need to be corrected according to

$$F_{actual} = \frac{\alpha_{IMF} F_{measured}}{1 - F_{measured}(1 - \alpha_{IMF})} \qquad \text{Eq. S7}$$

where α_{IMF} designates the instrumental mass fraction factor, which can be determined by measurement of a reference material. For NanoSIMS analysis of biomass, certified reference materials are not available yet. Measurements on microbial cells from marine sediments with natural isotopic composition that we conducted within the same analysis run yielded a ^{2}H isotope content of 0.012 +/- 0.003 at% (mean, +/- 1SD), which is well within the range of natural variation and exactly matches the value previously determined for membrane lipids of prokaryotes from a similar sediment[11]. As such, we excluded IMF and used the measurement value from the dead control as reference value in the calculation of biomass generation rates and replication times.

NanoSIMS is an element specific, isotope selective single cell analysis technique which means that NanoSIMS delivers the average ^{2}H content of a single cell. In contrast, lipid based SIP, which has also been applied for investigation of hydrogen assimilation in conjunction with heavy water labeling[2,3,7,11,12] exhibits compound specificity for fatty acids combined with determination of their isotopic composition through coupling to isotope ratio mass spectrometry (IRMS). Accordingly, lipid-based SIP can provide average anabolic activity rates of microbial communities though with particular focus on cellular membranes. Kopf *et al.*[2] conducted correlative analyses of NanoSIMS versus gas chromatography (GC) - pyrolysis - IRMS on bacterial cell cultures after heavy water labeling at distinct ^2H_2O concentrations and harvest in the mid-exponential phase. Prior to NanoSIMS analysis, cells were fixed in 1% formaldehyde solution for 2 hours at room temperature. They found a linear relationship between the average NanoSIMS (NS) single cell values and the measurement values from cellular fatty acid extracts (FA) in the range from natural isotopic abundance to 0.7 at% ^{2}H isotope content. For our purpose, their results can be expressed as

$$F_{labeled}^{NS} - F_{n.a.}^{NS} = s\left(F_{labeled}^{FA} - F_{n.a.}^{FA}\right) \qquad \text{Eq. S8}$$

where $F_{n.a.}$ and $F_{labeled}$ represent the ^{2}H content determined on isotopically unlabeled (natural isotope abundance) and isotopically labeled cells. s refers to the slope of the regression line, which was determined as 0.67 for cells of *Pseudomonas aeruginosa* and 0.59 for cells from *Staphylococcus aureus*, each chosen as representatives for Gram-negative and Gram-positive organisms, respectively. In other words, the ^{2}H content of the cells determined by NanoSIMS was 33% and 41% lower than obtained by FA analysis over the entire measurement range. This finding can be discussed in multiple aspects.

First, due to its element-specificity, NanoSIMS is sensitive to contamination by any kind of hydrogen containing compounds with natural isotope abundance introduced either by sample preparation or contained in the residual gas of the analysis chamber. FA analysis is more robust in this context since none of the reagents typically applied in sample preparation contains lipids. The potential influence of the residual gas is difficult to judge since the base pressure in the NanoSIMS analysis chamber was not specified in the publication of Kopf *et al.*[2] and further depends on the acquisition parameters (in

particular the probe current and scanning speed). The effect of sample preparation on NanoSIMS isotope composition measurement values has been investigated and quantified[13,14,15,16,17] but data for deuterium are scarce. IRMS bulk measurements have shown that PFA fixation leads to 8.2 +/-2.0% label dilution in ^{13}C labeled microbial cells[17]. Formaldehyde is an alkylating agent[18], leading to the addition of the hydroxymethylene group ($-CH_2OH$) to many functional groups of proteins or, upon extended exposure, a covalent cross-linking between individual proteins via methylene ($-CH_2-$) bridges[19]. Based on the H:C stoichiometry of these functional groups (i.e. 3:1 and 2:1) the observed ^{13}C label dilution corresponds to an expectable relative decrease of the ^{2}H label content of up to 25 +/-6%. In our study, cells were additionally stained with DAPI after fixation with 4% (v/v) formaldehyde solution for 1.5 hours at room temperature. According to its elemental composition ($C_{16}H_{15}N_5$), DAPI staining should lead to a further decrease in the NanoSIMS - determined deuterium content, depending on the abundance of nucleic acids, which also depends on the cell cycle. Interestingly, Meyer *et al.*[17] did not observe deuterium label dilution by DAPI staining, but up to 8% for ^{13}C. Utilizing the same approach as for PFA fixation, the cumulative effect of PFA fixation and DAPI staining on the decrease in the cellular deuterium content would amount to around 33% which is close to the values reported by Kopf *et al.*[2]. The remaining discrepancy may be due to a relatively higher deuterium enrichment of lipids than other macromolecules[20]. We decided to correct our NanoSIMS measurement values relative to FA analysis to accomplish comparability with lipid-based SIP. By definition of a correction factor (k), we calculated the values for F_{incub} and F_{ctrl} inferred from NanoSIMS analysis via

$$\left(F_{incub} - F_{ctrl}\right) = k\left(F_{incub}^{NS} - F_{ctrl}^{NS}\right) \qquad \text{Eq. S9}$$

with k corresponding to the inverse slope in the regression lines determined by Kopf *et al.* [2] (Eq. S 8), *i.e.*

$$k = s^{-1} \qquad \text{Eq. S10}$$

Insertion of Eq. S9 in Eq. S6 and solving for $\overline{\mu}$ finally yields the analysis function for inference of single cell biomass generation rates from NanoSIMS measurement data:

$$\overline{\mu} = -\ln\left(1 - \frac{k\left(F_{incub}^{NS} - F_{ctrl}^{NS}\right)}{a_w F_{D2O} - F_{H2O}}\right) / t_{incub} \qquad \text{Eq. S11}$$

(see equation (5) in **Methods**).

It is evident that k and a_w both have an impact on calculated biomass generation rates (Eq. S11) and cellular replication times (Eq. S2, Eq. S4). We defined two extreme case scenarios to cover the entire range of possible values. The lower bound for $\tau_{replication}$ results from selection of a_w = 0.28 and k = 1.69, the upper bound from a_w = 0.79 and k = 1.49. Since the variation is dominated by a_w, we designated the scenario referring to the lower bound as ’obligate chemoautotrophy‘ and the scenario referring to

the upper bound as ’obligate heterotrophy‘. For evaluation of the dynamics of anabolic activity in response to rehydration, the mean values of both parameters were chosen, *i.e.* $a_w = 0.54$ and $k = 1.59$ (**Supplementary Fig. 10** and **Supplementary Fig. 11**). We considered this as an appropriate approach to characterize the behavior of the entire community over the time series of incubations and note that the results were equivalent under application of the parameters chosen for ’obligate chemoautotrophy‘ and ’obligate heterotrophy‘ (**Fig. 2e**).

Experimental evaluation of ^2H dilution by the cell detachment and concentration procedure based on pure culture experiments

To ensure that we did not underestimate the rate of biomass generation and thus overestimated inferred replication times, and consequently concluded slow growth in biocrusts cells, by additional dilution of the ^2H label through the applied cell detachment and concentration procedure, we experimentally tested this effect with two heterotrophic strains. These were chosen because the replication times considering a heterotrophic physiology were the shortest in our estimations (**Fig. 2e,f**) and we wanted to confirm that we did not underestimate microbial growth during short rain events. The obtained values were then compared with the values obtained from the literature as described above. We chose *Rubrobacter radiotolerans* DSM 5868 (purchased from the DSMZ) as a closely related cultured representative of dominant members in the investigated biocrust microbial community. Further, we chose a *Sphingomonas* sp. strain (designated strain X13) that was isolated from Negev Desert biocrusts. The closest living relative being *Sphingomonas echinoides* ATCC 14820 (~99.5% sequence identity based on 1000 bp). Thus, both strains represent two major taxa (Actinomycetota and Alphaproteobacteria) that are commonly found in Negev Desert biocrusts. Cells were grown in R2A broth (Himedia R2A Broth, neoLab, Germany), aerobically at room temperature (22°C) in ^2H_2O (Sigma Aldrich, 99.9 atom % D, lot # STBJ4150), which was diluted to a final concentration of 30% (v/v). Medium was filter-sterilized by a PES type filter with a pore size of 0.2 µm. *Sphinomonas sp*. strain X13 was transferred twice in labelled liquid medium, while *Rubrobacter radiotolerans* DSM 5868 was grown once in labelled liquid medium. All cells were harvested in stationary phase and concentrated at 5000 g and 4°C for 20 minutes (5430R centrifuge, Eppendorf, Austria) to reduce shearing forces acting on the cells. The supernatant was discarded, and the cells were washed with non-labelled R2A medium and PBS buffer to remove the isotopically labelled medium. Cells were then carefully resuspended in PBS buffer. For preparing the controls, cells were neither fixed nor nucleic acid-stained but were directly concentrated on gold-coated polycarbonate filters and washed with approximately 100 mL 0.5 x PBS and 10 mL ultrapure water. To reduce the osmotic pressure of unfixed cells and preserving their cell integrity as best as possible, we used diluted PBS for the washing and only in a last step ultrapure water to reduce potential salt crystallization on

the filters during the air drying. The cell integrity was checked on separate filter pieces by nucleic acid staining (1 µg/mL 4’,6-diamidino-2-phenylindol, DAPI) and scanning electron microscopy (JEOL IT300). For testing the effect of PFA fixation and the cell detachment and concentration procedure on the isotopic content of our samples, an aliquot of the grown cells was harvested and washed with non-labelled R2A medium and PBS buffer. Washed cells were fixed in 4% PFA solution overnight at 4°C. After fixation, the cells were washed three times (once in PBS and twice with ultrapure water) to remove the remaining PFA. After the last washing step, the supernatant was discarded, cells were resuspended in 200 µl PBS, and 5 mL cell-detachment solution, used for the biocrust samples to separate cells and soil particles, was added. This solution contained 0.35% w/v polyvinylpyrrolidone (PVP), 0.5% v/v Tween20 and 3 mM sodium pyrophosphate. This cell suspension was incubated at room temperature for 30 minutes as described for the biocrust samples, and subsequently, cells were concentrated on gold-coated polycarbonate filters and washed with approximately 100 mL ultrapure water. The filters were air dried and stained with 1 µg/mL 4’,6-diamidino-2-phenylindol (DAPI) for 7 minutes followed by a last washing step with ultrapure water (~ 15mL).

NanoSIMS measurements were performed identically as applied in the analysis of the biocrust samples (see “NanoSIMS analysis” in **Methods**) with the exception that the first high energy Cs^+ ion bombardment during pre-conditioning was conducted to a fluence of 5×10^{14} ions per cm^2. Measurement data were exclusively acquired in the low current (LC) mode providing optimum spatial resolution, the determined values of the ^2H content are displayed in **Supplementary Fig. 2**.

The extent of ^2H label dilution was quantified by calculation of a dilution factor (DF) according to the formula published by Woebken *et al.*[14] and Meyer *et al.*[17], which reads

$$\mathrm{DF} = \frac{a_f - a_i}{a_{add} - a_i} \qquad \text{Eq. S12}$$

where a_i and a_f refer to the ^2H content measured in untreated cells (here, the control) and cells that were subjected to the entire sample preparation procedure including cell detachment, respectively. a_{add} stands for the ^2H content of isotopically unlabeled substances deposited in the cells by sample preparation. The exact ^2H content of the compounds used in sample preparation is unknown, but the data published by IUPAC suggest that it is in the range from 0.0026 at% to 0.0155 at%. In the calculation of DF, we applied the mean value being 0.0142 at%, affected with an uncertainty ($\Delta a_{add.}$) of +/- 0.0065 at%. The uncertainty in the values of a_i and a_f were inferred from the standard deviations of the measurement data distributions $(\sigma_{a_i}$ and $\sigma_{a_f})$ after confirming a normal distribution by the asymptotic one-sample Kolmogorov-Smirnov test at a level of significance of $p > 0.05$. Based on the error propagation theorem, the uncertainty of the DF values was calculated via

$$\Delta_{DF}=\frac{1}{(a_{add}-a_i)^2}\sqrt{(a_f-a_{add})^2\sigma_{a_i}^2+(a_{add}-a_i)^2\sigma_{a_f}^2+(a_i-a_f)^2\Delta_{a_{add}}^2} \qquad \text{Eq. S13}$$

which is equivalent to the formula used by Meyer *et al.*[17], but being extended by the term which is taking into account the uncertainty of $a_{add.}$. Utilizing Eq. S12 and Eq. S13 yielded DF = 0.23 +/- 0.30 for single cells of *Rubrobacter radiotolerans* DSM 5868 and DF = 0.41 +/- 0.08 for single cells of *Sphingomonas* sp. strain X13. We note that the indicated uncertainty in the single cell data retrieved from *Rubrobacter radiotolerans* DSM 5868 is rather high, which emerges from the spread in the ^{2}H content values already detected in the untreated cells. This is most likely due to the circumstance that these cells were only grown once in the isotope-labeled liquid media (*i.e.* without further transfer). However, the high number of analyzed cells (**Supplementary Fig. 2**) enables a high-quality estimation of the central value and its variation caused by the treatment. In this context, the standard error of the mean ($\sigma/\sqrt{n}$) is the more meaningful measure of uncertainty, yielding +/- 0.02 for *Rubrobacter radiotolerans* DSM 5868 and +/-0.01 for *Sphingomonas* sp. strain X13. Accordingly, the dilution of the ^{2}H label brought about by sample preparation can be expected as 23 +/- 2% and 41 +/- 1%, respectively. We therefore concluded that the NanoSIMS correction factor applied in the scenario 'obligate heterotrophy', which is based on the 41% lower NanoSIMS - determined ^{2}H content values relative to lipid-SIP found by Kopf *et al.*[2], is realistic and that the long replication times of the biocrust cells in our study are not the result of dilution in the ^{2}H label by our sample preparation procedure including cell detachment.

Limit of Detection (LOD) and its consequences on inference of biomass generation rates

As specified above, our ^{2}H content measurement values of individual cells from the dead control showed a standard deviation of 0.006 at%. This means that, based on a 3σ criterion for the LOD (corresponding to a confidence level of $p < 0.00135$), the minimum ^{2}H enrichment of a single cell needs to be higher than 0.018 at% (relative to the dead control) for being detected as anabolically active, which leads to a constriction in the minimal determinable biomass generation rate. According to Eq. S11, the boundary value depends, in addition to the k and a_w values, also on the incubation time. **Supplementary Table 4** shows the respective values and illustrates that a cell exhibiting an obligate heterotrophic physiology needs to divide within less than 24 days for being detected as active in a 3-hour incubation. On the upper end, a cell from a chemoautotroph organism in the physiological state of sole maintenance may take up to 2.5 years in complete regeneration of its cellular constituents for being detected as active in a 24-hour incubation.

As outlined in the main text (**Methods**), the minimum expectable random measurement error in determination of an isotopic composition by NanoSIMS imaging is determined by the statistical

uncertainty in single ion counting. This means that the precision achievable in measurement of a single cell depends on the total number of secondary ions detectable from the respective cell. For the sake of illustration, let us assume a ^{2}H content measurement in which entire cells with similar morphology are sampled (*i.e.* complete sputter-erosion of the cellular material during the measurement process). Under these conditions, the total number of $^{1}H^{-}$ and $^{2}H^{-}$ secondary ions registered per cell depends on the total elemental hydrogen content of each cell. If the biomass density is independent from the cell cycle, it follows that the measurement precision scales with cell size. In other words, the analytical uncertainty in the measured ^{2}H content value of a small cell is higher than for a large cell. We have taken this source of variable analytical uncertainty into account by implementation of the second criterion for classification of ^{2}H enrichment, *i.e.* individual cells were only considered as significantly enriched, if the random measurement error ($3\sigma_{Poisson}$, corresponding to $p < 0.00135$) was smaller than the difference between the ^{2}H content of the interrogated cell and the mean of the single cells from the dead control (see equation (1) in **Methods**). From the viewpoint of significance testing, application of this second criterion enhances the certainty in detection of anabolic activity for each individual cell. However, it simultaneously enhances the risk that measurement values from cells are rejected which were anabolically active, resulting in overestimation of the anabolic activity on the community level. *Vice versa*, embracing all measurement values, regardless of the level of isotopic enrichment, results in underestimation of anabolic activity through inclusion of inactive cells.

With respect to characterization of the dynamics of resuscitation, the decisive question is in how far the central value of the distribution of the calculated single cell biomass generation rates varies over the incubation time. For the sample exhibiting the lowest overall deuterium enrichment (3-hour incubation), only 68% of all sampled cells passed both criteria for significant isotopic enrichment. By extended incubation, the fraction of significantly enriched cells continuously increased, reaching 95% after 24 hours of incubation. **Fig. 2e** shows that the calculated biomass generation rates of the community are balanced relative to the variance, if judged on the median of the significantly enriched cells. If inferred from the median of all measurement values, the biomass generation rates are lower with a drastic offset for the sample obtained from 3 hour of incubation (**Supplementary Fig. 10d**). As expectable from the increasing fraction of significantly enriched cells, the discrepancy decreases by enhancement of the incubation time. This rises the basic question which of the median values most accurately describe the average anabolic rate of the entire community. Utilizing the high number of analyzed cells ($n > 210$ per sample), we plotted relative frequency distributions, which showed a similar shape for each sample, *i.e.* a sharp peak with a right-tale towards higher biomass generation rates appearing as a shoulder for the samples from 3, 6 and 24 hour of incubation (**Supplementary Fig. 11**). Such a similarity offers the opportunity to extrapolate the information gained from data sets with high analytical certainty to data sets containing a substantial number of measurement values

close, or even below, the LOD. For a more precise characterization of the frequency distributions, we conducted curve fitting based on accumulation of Gaussian probability density functions ('peaks') with manual selection of the mean values and standard deviations. **Supplementary Fig. 11** shows that each of the frequency distributions could be fitted by three consecutive peaks with increasing standard deviation at simultaneously decreasing relative areas. The central value of the fitted distribution was calculated as the area-weighted mean of the three peaks, which was slightly above the median obtained from the significantly enriched cells except for the 3 and 24 hour incubation, which showed a close match (**Supplementary Fig. 10d**). Most importantly, the trends displayed for the biomass generation rates over the incubation time are similar, which corroborates that the median of the enriched cells provides, in our study, a realistic view on the dynamics of anabolic activity of the community in response to rehydration (**Fig. 2e**).

Supplementary Note 2

<u>Bulk metatranscriptome analysis:</u>

Bulk transcriptome composition and representation by the MAG-resolved fraction

When mapping RNA reads to metagenomes, we were able to assign between 12 and 30% of reads (19% on average) to metagenomic contigs. On average, 15% of transcripts were assigned to MAGs from Meier *et al*. 2021 while further 4% were assigned to unbinned contigs (**Supplementary Fig. 12**). The unbinned contigs largely derive from poor genomic bins with low completeness and/or high level of duplication of single-copy marker genes. Furthermore, they contain short contigs that could not be assigned to any genomic bin by the binning algorithms.

We classified the reads that did not map to the metagenome with Kaiju classifier (v.1.9.2)[21]. Between 51% and 75% (61% on average) of the mRNA reads could not be assigned to any organism or taxonomically classified in any way (**Supplementary Fig. 12**). While the phenomenon of large fraction of unclassified reads is observed even in datasets stemming from mock communities with known genomic content[22]. The unclassified reads could also belong to yet unknown groups of environmental viruses or desert soil inhabiting eukaryotes (*e.g*. microalgae, fungi, animals, plants) not represented in public sequence databases. The metagenomic rRNA data has indicated the presence of eukaryotic genes in the extracted nucleic acids[23]. Although their proportion was rather low in the DNA data (5% on average), their large cells would likely contain high amounts of RNA when active and significantly contribute to the transcriptomes.

The unmapped reads that could be taxonomically classified by Kaiju (11-29%, 19% on average, **Supplementary Fig. 12**), largely belonged to the same taxa as represented by the MAGs such as *Microcoleus* (up to 8.4%, 3.2% on average), *Rubrobacter* (up to 2.7%, 1.5% on average), *Oscillatoria* (some members of the taxa have been reclassified to *Microcoleus*, see Genome Taxonomy Database)(up to 2.1%, 1.2% on average), *Tychonema* (reclassified to *Microcoleus*, up to 1.4%, on average 0.8%), *Coleofasciculus* (up to 1.1%, 0.5% on average), *Blastococcus* (up to 3.1%, 0.4% on average), other *Cyanobacteria*, *Actinobacteria*, *Alphaproteobacteria*, *Bacteroidota, Gemmatimonadota*. Significant taxa that were detected in the unmapped transcriptome but are not represented by the MAGs were *Nitrosophaeria* archaea (up to 1.7%, 0.4% on average), *Bacilli* (up to 0.9%, 0.3% on average), and *Vibrio* (up to 0.9%, 0.4% on average). *Nitrososphaeria* and *Bacilli* sequences were present in the 16S rRNA gene data and represent autochthonous low abundant soil crust taxa, while *Vibrio* sequences were not previously detected in these crusts. An explanation for the presence of *Vibrio* could be misclassification of short reads by Kaiju. Short reads are classified by aligning translated sequence fragments to databases with reference genomes. One should consider that i) many protein sequences do not have a strong phylogenetic signal, ii) only a short fragment of a protein sequence is aligned, iii) many environmental taxa are not represented in the reference databases with full genomes, iv) model organisms, medically or biotechnologically relevant taxa are overrepresented in the database and are more likely to receive random false positive hits.

Taken together, we consider the MAG-centered data analyzed for this manuscript representative of the biocrust microbial community, even though it clearly does not cover it in its entirety. It covers a variety of dominant microbial taxa with a range of different metabolisms from photoautotrophic to chemolithoheterotrophic and complex organic matter degraders.

Clustering of samples by transcription patterns of microbial populations

As described in the main text, hierarchical clustering as well as NMDS ordination distinguished three major sample groups: dry samples, early hydrated, main hydrated. The significant differences between the groups were confirmed by ANOSIM test (**Fig. 3a**). However, in both hierarchical clustering and NMDS plot, it is evident that the dry samples can be sub-divided further into time point 0-hour samples and dry samples at the end of the experiment (T39 and T55).

On the one hand, the fact that all dry samples are more like each other than to hydrated samples indicates that the microorganisms do return to a similar transcriptional state after the experiment as they were before the rehydration. On the other hand, the distinct differences between the T0 and T39/T55 samples show that these two states are not identical. This discrepancy almost certainly results from the difference of incubation conditions in the laboratory and a natural rain event in the desert.

Although we delayed and slowed down desiccation by incubating the crusts in closed petri-dishes and removing the parafilm only after 24 hours, the desiccation in our climate chamber was most likely still more rapid (< 24 hours) than in the field (several days). Thus, the shortened desiccation window might have had an influence on the transcription pattern in the desiccated state. Furthermore, although our climate chamber has a powerful light source (500 µmol × m^{-2} × s^{-1}), it cannot reach the field intensities of several thousand µmol (cloudy day). In our incubations, we were also missing flora (e.g. shrub plants and their roots) and fauna which might interact with the microbial community and impact its transcriptional response. It is thus not surprising that transcriptional profiles of microorganisms that have naturally desiccated in the field (T0) are different from profiles after a desiccation in the lab (T39 and T55). The advantages of our laboratory set-up as opposed to a field study are the controlled start and end as well as the pre-screened microbial composition of the crust. In a field experiment one would have to wait for a natural rain event or create set-ups reducing the light, temperature and desiccation to simulate rain in the field on a hot desert day. In the field replicate crust patches can only be selected based on appearance. For our experiment the composition of different sampled patches was pre-screened by 16S rRNA amplicon sequences ensuring that they largely represent replicates of the same microbial community. Lastly, the regular immediate freezing of the samples in liquid nitrogen over the course of 55 hours would have been difficult to realize in the field. In our experience samples frozen in liquid N_2 and stored at -80°C provide a superior quality and quantity of RNA compared to samples preserved with fixatives like RNA-later. The mentioned differences would have led to different biases in a field set-up.

Patterns detectable in the bulk dataset

An overall change in gene transcription of the microbial community was evident in the bulk transcriptome profiles, with transcripts having a more even distribution in the hydrated biocrusts (Inverse Simpson Index of evenness: 1646 ± 314) compared to dry biocrusts (Inverse Simpson Index of evenness: 820 ± 240) (**Supplementary Fig. 13a**). Based on assigned COG categories, we observed a pattern among the different stages of the hydration. Genes related to transcription, translation, ribosomal structure and biogenesis, post translational modification, protein turnover and chaperones received a larger proportion of overall transcripts between 15 to 30 minutes after hydration (**Supplementary Fig. 13b**), while between 3 and 12 hours, transcripts related to carbohydrate transport and metabolism, secondary metabolites, energy production and conversion, cell motility and extracellular structures reached highest proportions. Transcripts assigned to the category “function unknown” comprised the largest fraction overall and were slightly more prevalent in dry samples.

Supplementary Note 3

<u>Testing stoppers for controlled atmosphere gas incubations:</u>

H_2 production stemming from butyl rubber and silicon stopper

Although previous reports suggest that butyl-rubber stoppers artificially produce large quantities of H_2 [24], our initial testing of in-house butyl-rubbers and silicone stoppers (**Supplementary Table 3**) did not reveal that same pattern (**Supplementary Fig. 9**). Bottles were sealed with synthetic air, harboring and initial atmospheric concentration of H_2 of approximately 0.5 ppmv (**Supplementary Fig. 9a**). After 10 days, the silicon-based stoppers had ca. 10-fold higher H_2 concentrations as compared to butyl-rubber stoppers. Even after three rounds of autoclaving the silicone-based stoppers in ultrapure water to 'clean' the stoppers of impurities, there was still a 7.5-fold higher concentration of H_2 in the 'autoclaved silicone' stoppers compared to the butyl-rubber ones (**Supplementary Fig. 9a**). Therefore, our H_2 consumption experiments were performed butyl-rubber stoppers. As gas samples are typically stored in samples bottles prior to measurement (*i.e.* 3 mL exetainer vials, IVA Analysentechnik, Germany), we also explored the different materials and pre-treatments which can be used to seal tubes, namely butyl rubber, silicon and NaOH-treated butyl rubber as suggested in[24]. Technical specifications and catalogue numbers can be found in **Supplementary Table 3**. The butyl rubber and silicone-based stoppers exhibited similar performances. The H_2 concentrations increased ca. 1.2-fold (butyl rubber) and 1.1-fold (silicone) after 3 days, and ca. 1.4-fold (in both butyl rubber and silicone) after 6 days (**Supplementary Fig. 9b**). In contrast, the NaOH-treated butyl rubber stoppers increased 1.1-fold and 2.5-fold after 3 and 6 days, respectively (**Supplementary Fig. 9b**). In this study, we performed direct injections to measure headspace H_2 gas.

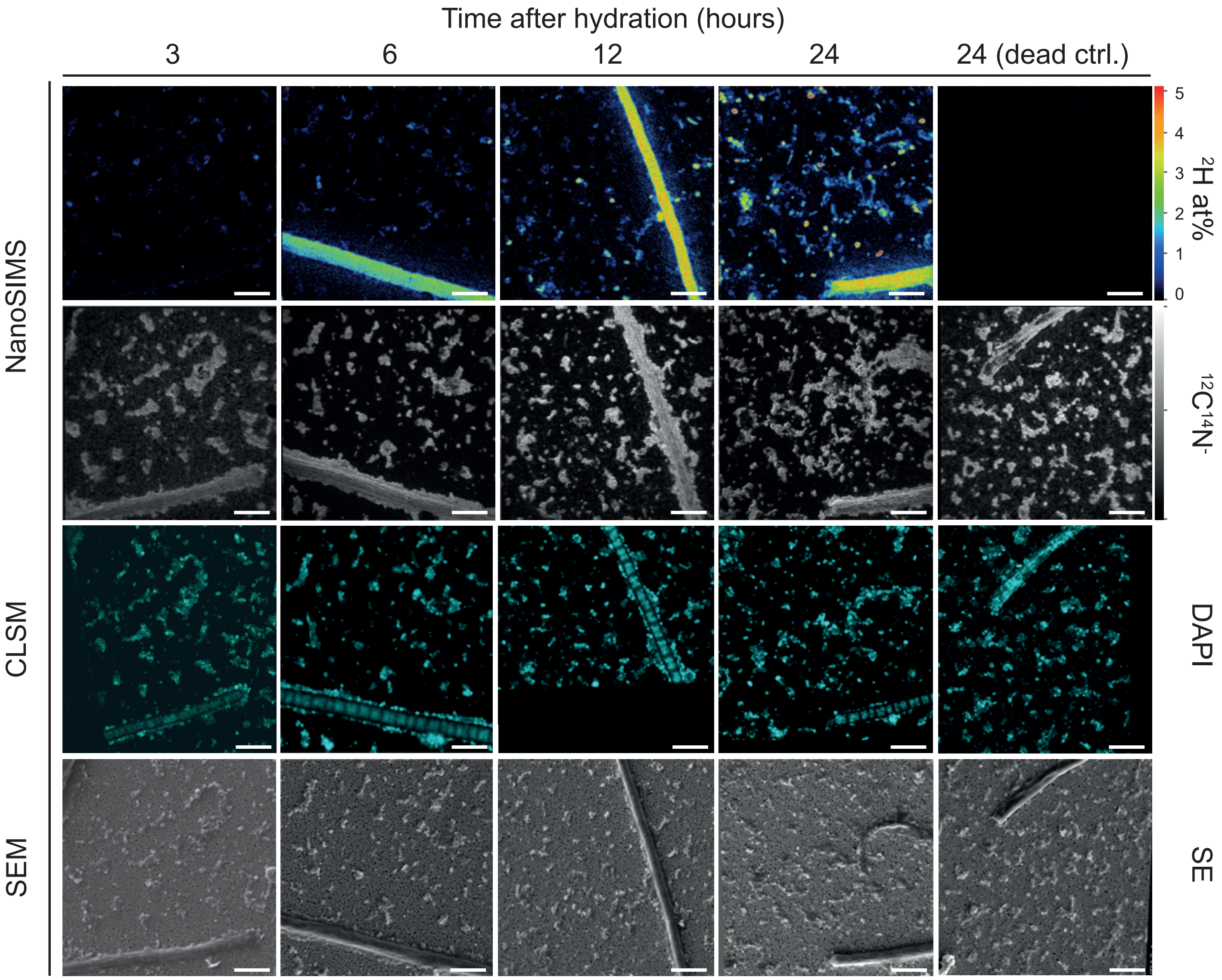

Supplementary Fig. 1. High-resolution NanoSIMS imaging combined with correlative confocal and scanning electron microscopy images.

The first row displays the average isotopic content of deuterium (^{2}H) of single cells and cyanobacterial filaments in at% after 3, 6, 12 and 24 hours of exposure to 30% (v/v) $^{2}H_2O$ including a negative (dead) control. The control was fixed by 48-hours exposure to 4% (v/v) formaldehyde solution prior to 24 hours incubation with 30% (v/v) $^{2}H_2O$. The second row corresponds to the nitrogen signal of a single cycle in $^{12}C^{14}N^{-}$ counts. Fields of view were preselected via CLSM (third row) and SEM (fourth row) to enhance the through-put of NanoSIMS analysis. Cells were previous to confocal laser scanning microscopy (CLSM) stained with 4’, 6-diamidino-2-phenylindole (DAPI). Secondary electron emission (SE) detected with a JEOL IT300 scanning electron microscope (SEM) under high vacuum mode to define the morphology of cells. The contrast of SEM imaging was optimized by deposition of a thin gold coating on the samples after CLSM imaging. Scale bars correspond to 5 µm.

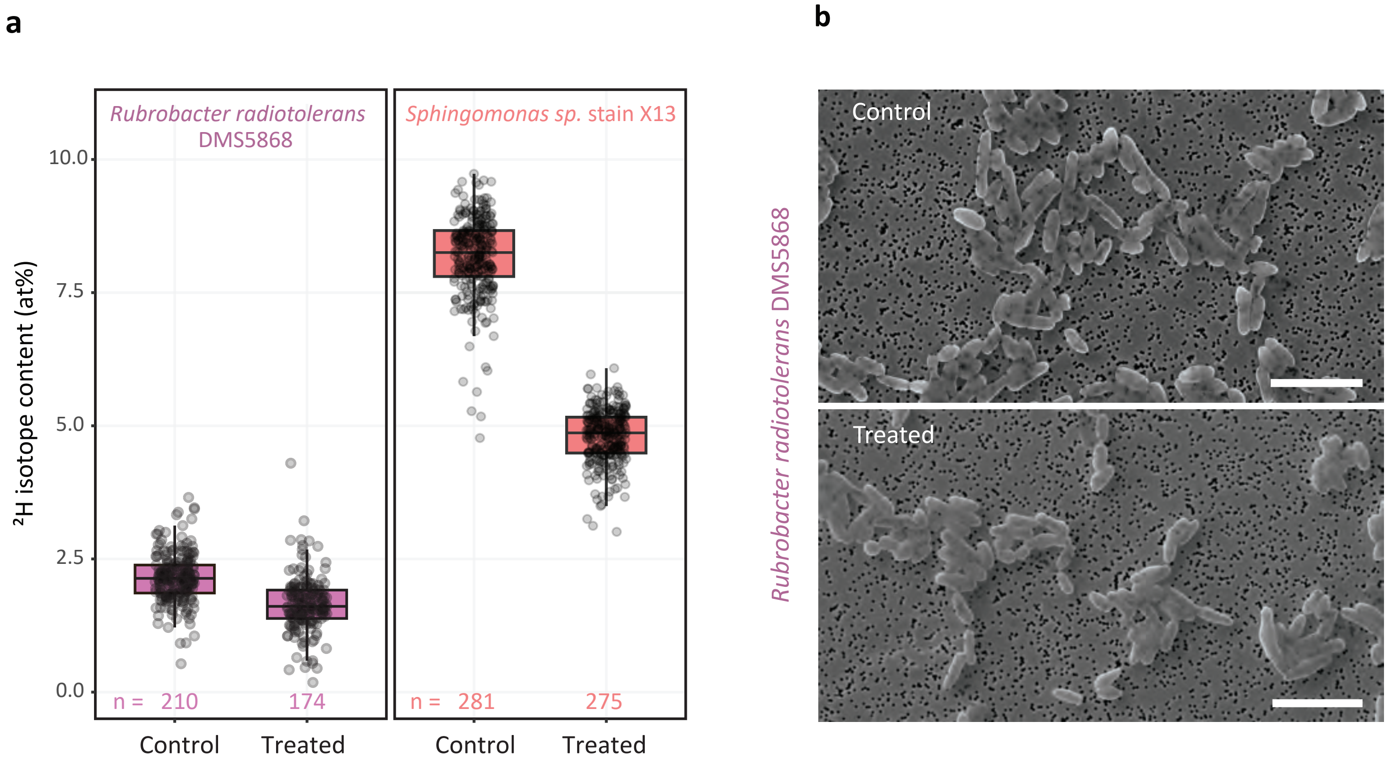

Supplementary Fig. 2. Influence of sample preparation on the ^2H isotope content of *Rubrobacter radiotolerans* DSM5868 and *Sphingomonas sp.* strain X13.

a, ^2H isotope content of single cells from two different bacterial strains (*Rubrobacter radiotolerans* DSM5868 and *Sphingomonas sp.* strain X13) grown in R2A medium containing 30% (v/v) ^2H_2O harvested in the stationary phase. Unfixed cells are referred to as “Control”, “Treated” designates cells fixed with 4% (v/v) paraformaldedyde subjected to a cell-detachment protocol and stained with 4’,6-diamidino-2-phenylindole (DAPI). n symbolizes the number of single cells analyzed by NanoSIMS. The boxes comprise the 2nd and 3rd quartiles with the horizontal line indicating the median. Whiskers maximally extend to 1.5 times the inter-quartile range. **b,** Scanning electron microscopy (SEM) images of *Rubrobacter radiotolerans* DMS5868 samples. Scale bars correspond to 5 µm.

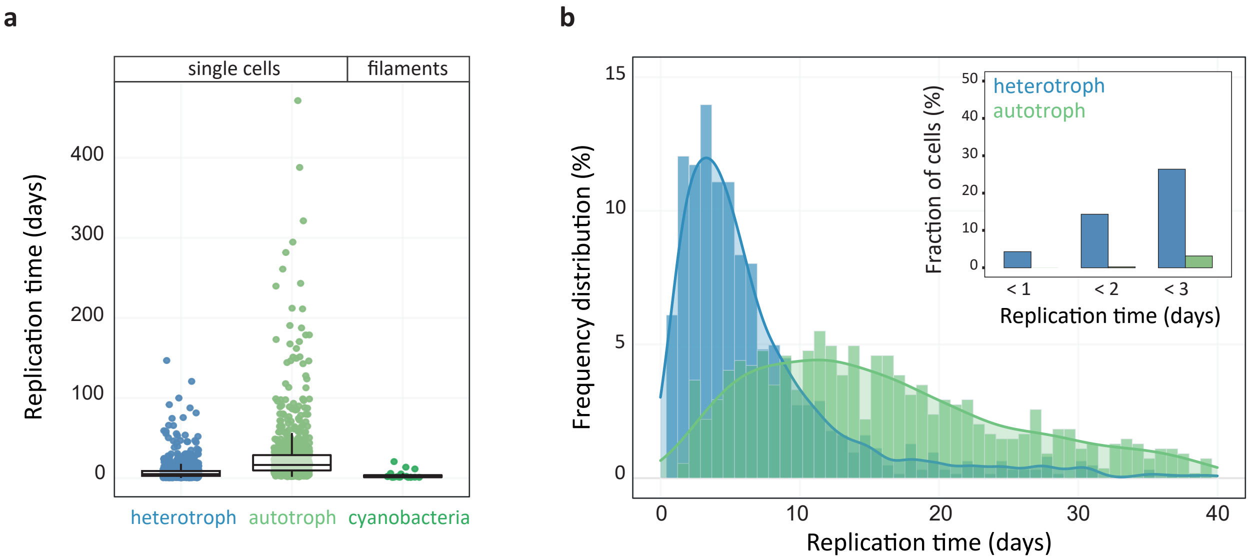

Supplementary Fig. 3. Estimated replication times based on the NanoSIMS detected ²H isotope content (at%) within single cells and cyanobacterial filaments.

a, Cellular replication times of single cells (based on the assumption of a heterotrophic or chemoautotrophic physiology) and of cyanobacterial filaments. For single cells, data acquired from incubations for 3, 6, 12 and 24 hours (n= 788) and for cyanobacterial filaments data acquired from 3, 12 and 24 hours incubations (n = 19) are included. The boxes comprise the 2nd and 3rd quartiles with the horizontal line indicating the median. Whiskers maximally extend to 1.5 times the inter-quartile range. **b**, Histogram visualizing the frequency distribution of the cellular replication times presented in panel **a**. Only replication times up to 40 days are displayed, representing 97% and 85% of all analyzed cells under the assumption of hetero- or chemoautotroph physiology, respectively. The inset shows the fraction of cells that are potentially able to divide within 1, 2 or 3 days.

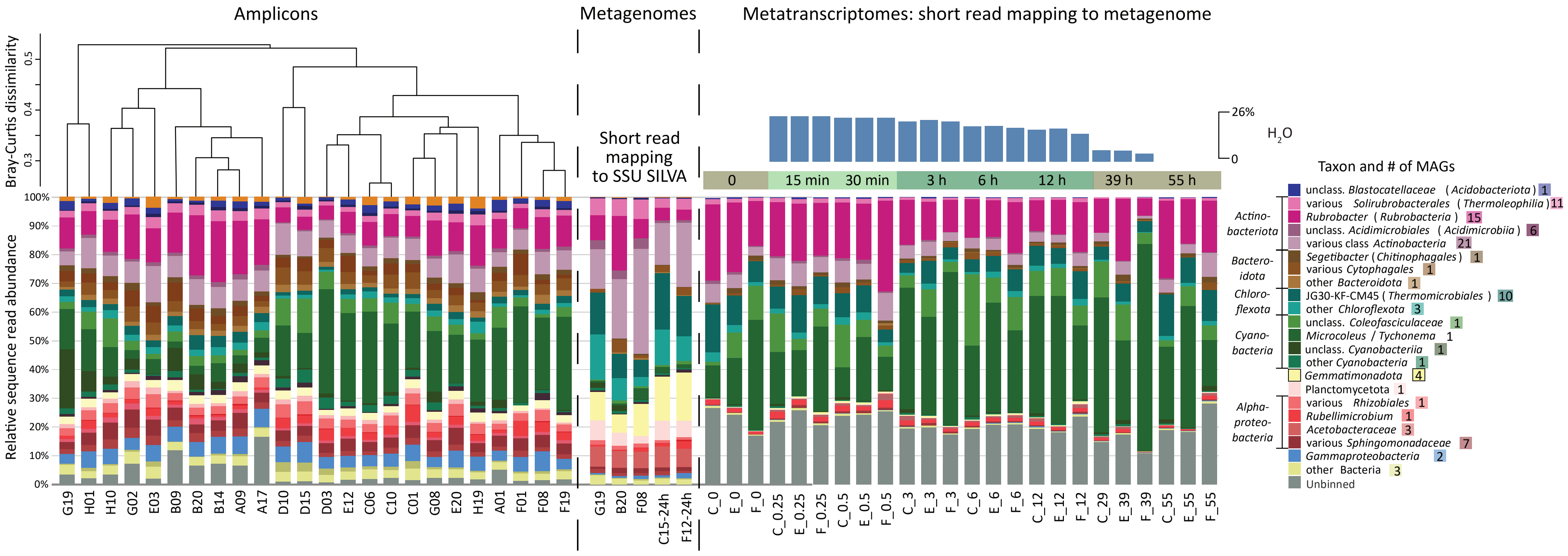

Supplementary Fig. 4. Microbial community composition in sampled dry biocrusts and rehydrated samples for metatranscriptome sequencing.
The community composition was determined by 16S rRNA gene amplicon (dry biocrusts) and metagenomic sequencing (dry biocrusts and crusts rehydrated for 24 hours) as described in Meier et al. 2021. Microbial community compositions were further investigated of samples undergoing the rehydration experiment. For analysis based on the metatranscriptomes, mRNA reads were mapped to the assembled metagenome and taxonomy was assigned based on the MAG they mapped to.

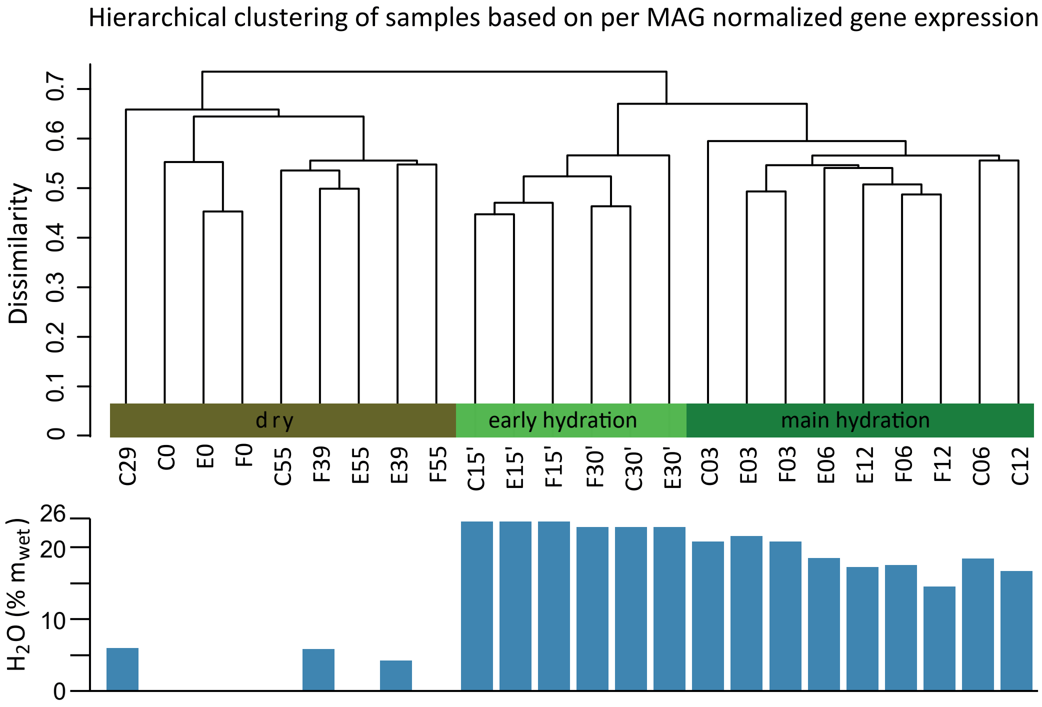

Supplementary Fig. 5. Hierarchical clustering of samples based on per-MAG-normalized relative transcript abundances. The average linkage clustering was performed in R based on Jaccard distance matrix calculated with the vegan package v. 2.5.4 (http://cran.r-project.org/package=vegan). Note, that the samples in the "main hydrated" cluster can not be differentiated further by specific time point. The different experimental phases are color coded: dry time points (brown), early hydration (light-green), main hydration phase (dark-green).

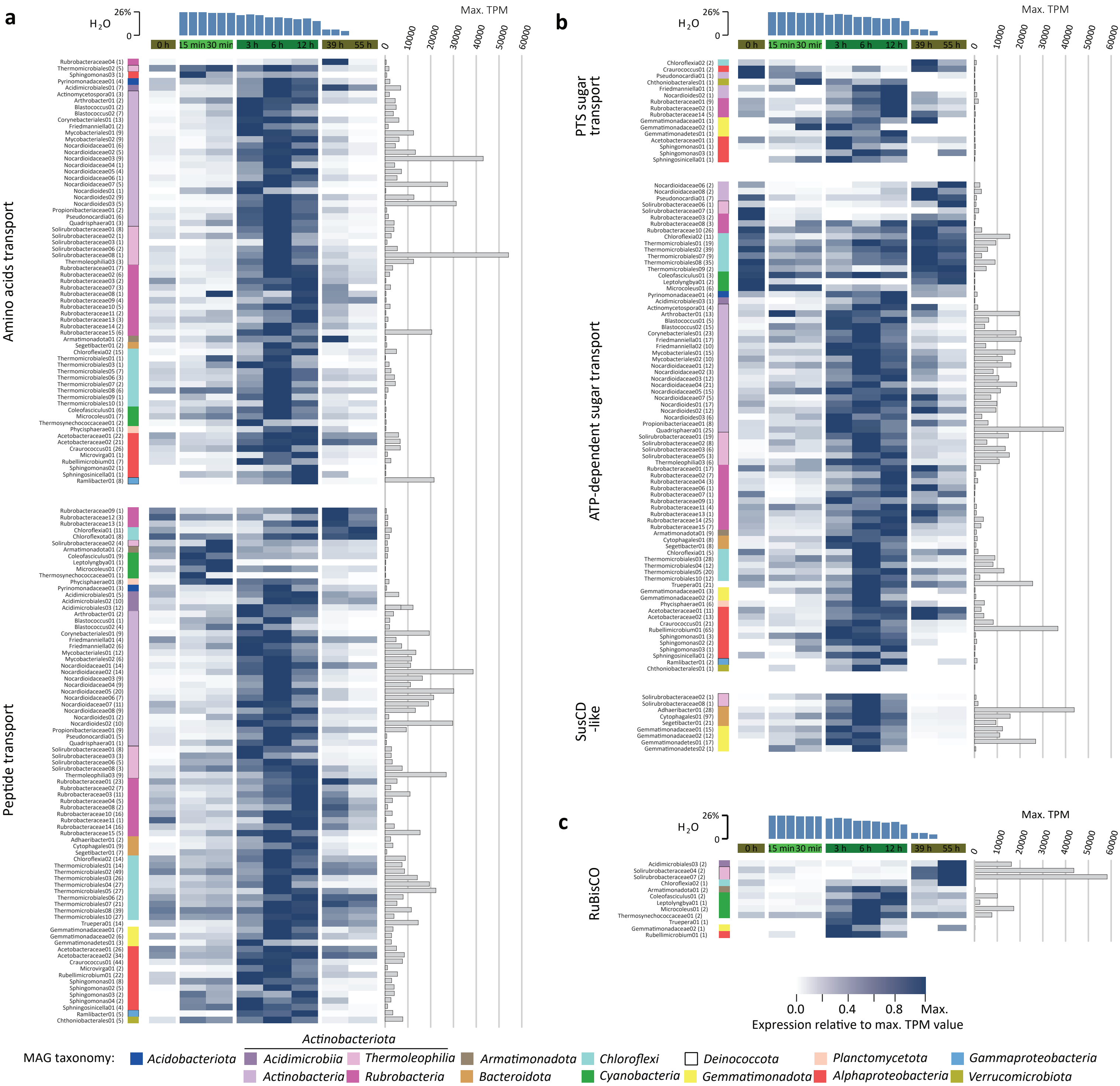

Supplementary Fig. 6. Relative transcript abundances of genes encoding carbon acquisition mechanisms.

a, Genes encoding transporters for amino acids and peptides; **b,** Genes encoding transporters for sugars and polysaccharides; **c,** Genes encoding ribulose 1,5-bisphosphat carboxylase (autotrophic carbon fixation). Only genes with a significant change in expression (DeSeq2 (Wald test) adj. $p < 0.05$) between subsequent time points (n=3 independent crust samples per time point) or between experiment phases (n=6 independent crust samples in early hydrated phase, n=9 for dry and main hydrated phase) are shown. Columns of the heat maps show individual time points of the time series (average values of three replicates). Effect sizes (as Log2-fold change) and Benjamini-Hochberg false discovery rate adjusted *p*-values (calculated with DeSeq2) for analyzed genes indicative of discussed metabolisms, can be found in Supplementary Data 3. Rows show transcripts attributed to a specific MAG. The numbers in brackets indicates how many genes were summarized per MAGs (encoding different subunits or multiple copies of the same gene). The highest color intensity indicates the time point where the respective transcript reached its highest proportion in a MAG's transcriptome. This maximum value is indicated in on the right in transcripts per million (TPM) (grey bars).

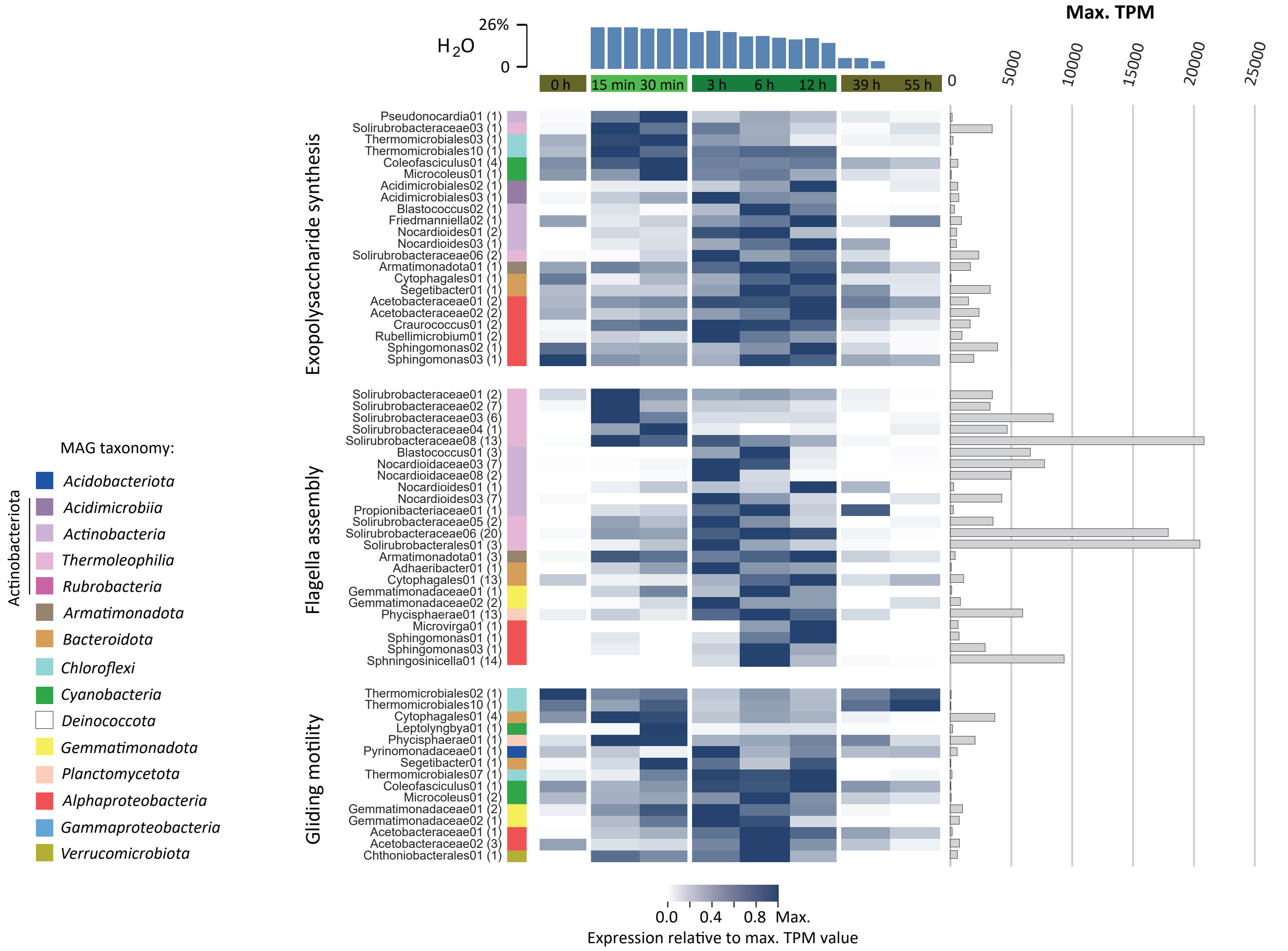

Supplementary Fig. 7. Relative transcript abundances of genes encoding exopolysaccharide synthesis and motility associated genes. Columns of the heat maps show individual time points of the time series (average values of three replicates). Rows show transcripts attributed to a specific MAG. The numbers in brackets indicate how many genes were summarized per MAGs (encoding different subunits or multiple copies of the same gene). Only genes with a significant change in expression (DeSeq2 (Wald test) adj. $p < 0.05$) between subsequent time points (n=3 independent crust samples per time point) or between experiment phases (n=6 independent crust samples in early hydrated phase, n=9 for dry and main hydrated phase) are shown. Columns of the heat maps show individual time points of the time series (average values of three replicates). Effect sizes (as Log2-fold change) and Benjamini-Hochberg false discovery rate adjusted *p*-values (calculated with DeSeq2) for analyzed genes indicative of discussed metabolisms, can be found in Supplementary Data 3. Rows show transcripts attributed to a specific MAG. The numbers in brackets indicates how many genes were summarized per MAGs (encoding different subunits or multiple copies of the same gene). The highest color intensity indicates the time point where the respective transcript reached its highest proportion in a MAG's transcriptome. This maximum value is indicated in on the right in transcripts per million (TPM) (grey bars).

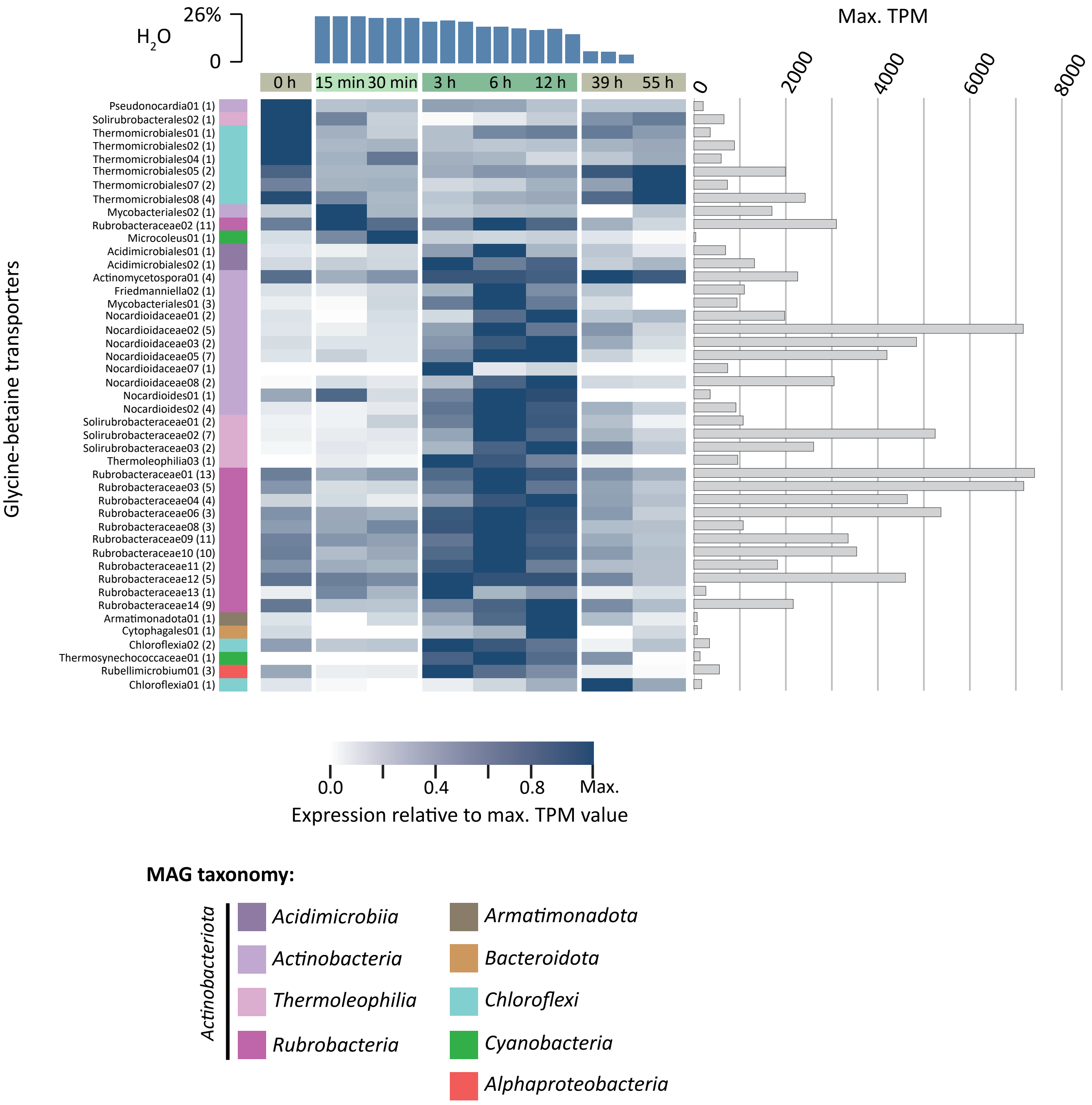

Supplementary Fig. 8. Relative transcript abundances of genes encoding glycine-betaine transporter genes.
Columns of the heat maps show individual time points of the time series (average values of three replicates). Rows show transcripts attributed to a specific MAG. The numbers in brackets indicate how many genes were summarized per MAGs (encoding different subunits or multiple copies of the same gene). Only genes with a significant change in expression (DeSeq2 (Wald test) adj. $p < 0.05$) between subsequent time points (n=3 independent crust samples per time point) or between experiment phases (n=6 independent crust samples in early hydration phase, n=9 for dry and main hydration phase) are shown. Columns of the heat maps show individual time points of the time series (average values of three replicates). Effect sizes (as Log2-fold change) and Benjamini-Hochberg false discovery rate adjusted *p*-values (calculated with DeSeq2) for analyzed genes indicative of discussed metabolisms, can be found in Supplementary Data 3. Rows show transcripts attributed to a specific MAG. The numbers in brackets indicates how many genes were summarized per MAGs (encoding different subunits or multiple copies of the same gene). The highest color intensity indicates the time point where the respective transcript reached its highest proportion in a MAG's transcriptome. This maximum value is indicated in on the right in transcripts per million (TPM) (grey bars).

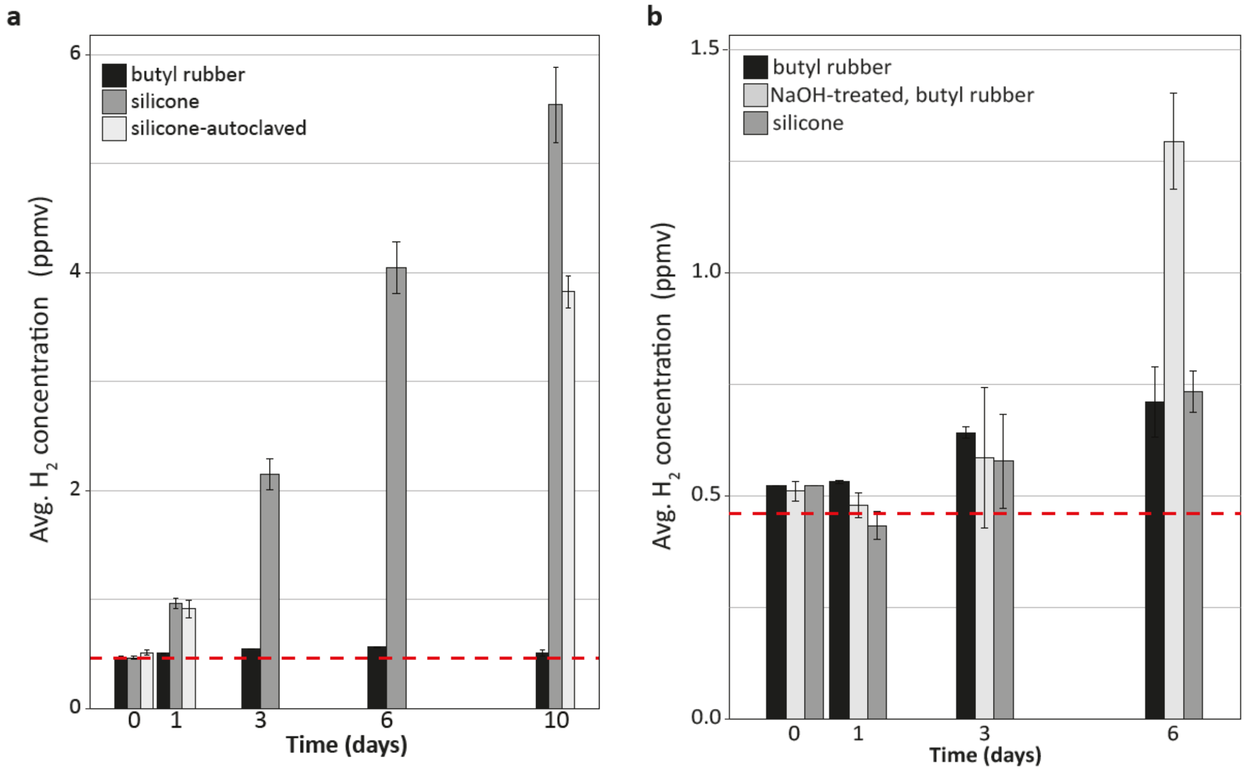

Supplementary Fig. 9. Hydrogen release stemming from stopper material.
Average H_2 production (+/- standard error) stemming from differing stopper material of serum bottles **a,** and exetainer vials **b,** over time. Red-dotted line represents atmospheric concentrations of H_2 (0.53 ppmv).

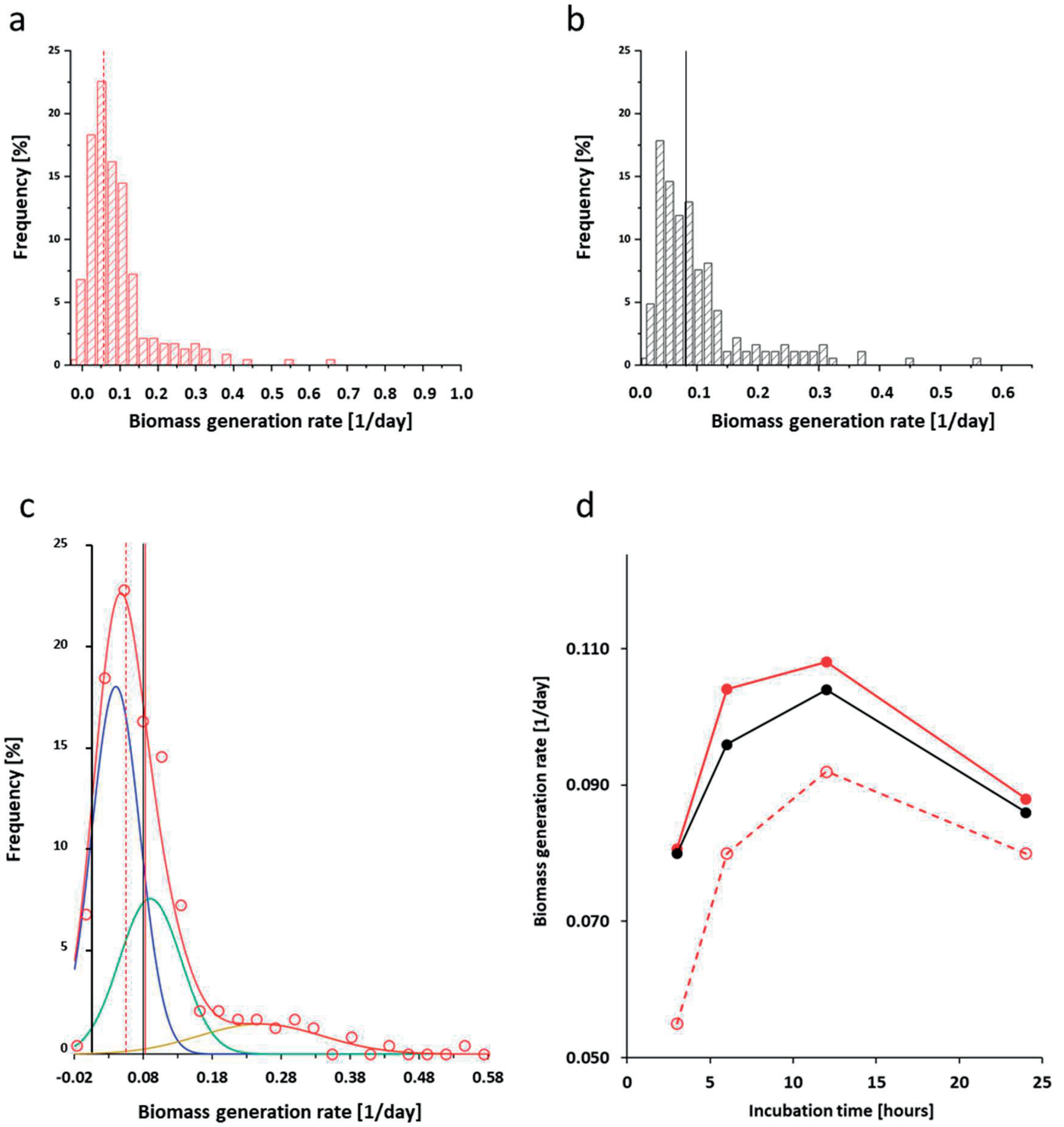

Supplementary Fig. 10. Statistical analysis of the NanoSIMS - inferred single cell biomass generation rates. The displayed data were obtained from the sample incubated for 3 hours. **a**, Frequency distribution of the single cell biomass generation rates based on all measurement data and **b**, selected for significant ^{2}H enrichment (p = 0.00135). **c**, Curve fit (red curve) of the frequency distribution of all single cell values (open circles), based on the accumulation of three Gaussian probability density functions (blue, green and orange curves) with each manually adjusted mean values and standard deviations. **d**, Estimated central values of the single cell biomass generation rate distributions as a function of incubation time. **a-d**, Red, dashed line: median of all analyzed single cells; black, solid line: median for significantly ^{2}H enriched cells; red, solid line: mean value obtained from curve fitting. The input parameters for calculation of generation rates (Eq. S11) were chosen as k = 1.59 and a_w = 0.54.

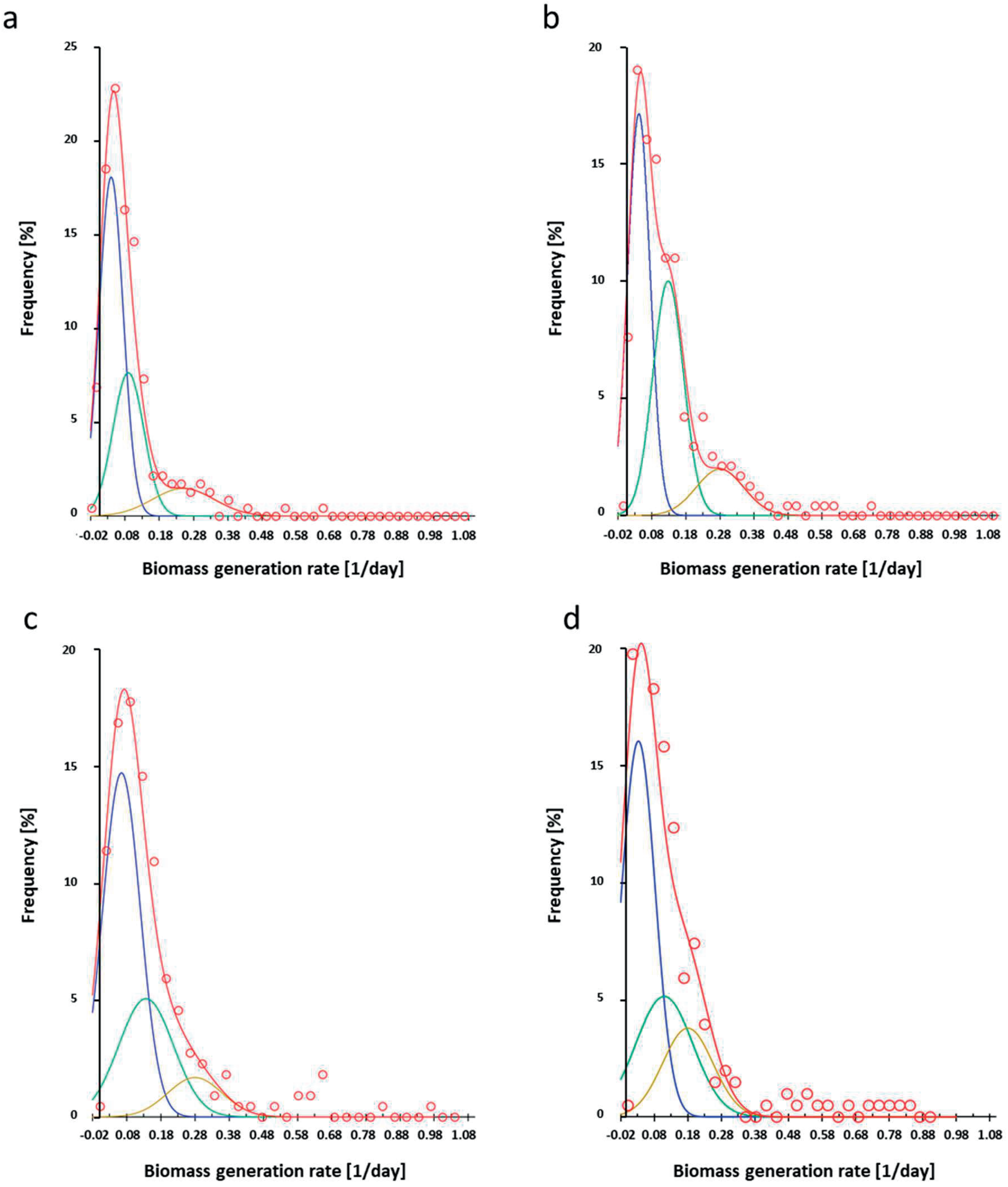

Supplementary Fig. 11. Comparison of the frequency distribution of single cell biomass generation rates.
Open circles indicate the observed frequency distribution of single cells values. Red curves display fitted curves based on accumulation of three Gaussian probability density functions (blue, green and orange curves) with each manually adjusted mean values and standard deviations. Incubation times and number of NanoSIMS - analyzed cells: **a**, 3 hours, n = 234; **b**, 6 hours, n = 234; **c**, 12 hours, n = 211; d, 24 hours, n = 242. The input parameters for calculation of the generation rates (Eq. S11) were chosen as k = 1.59 and a_w = 0.54.

Metatranscriptomes: short read mapping & taxonomic classification

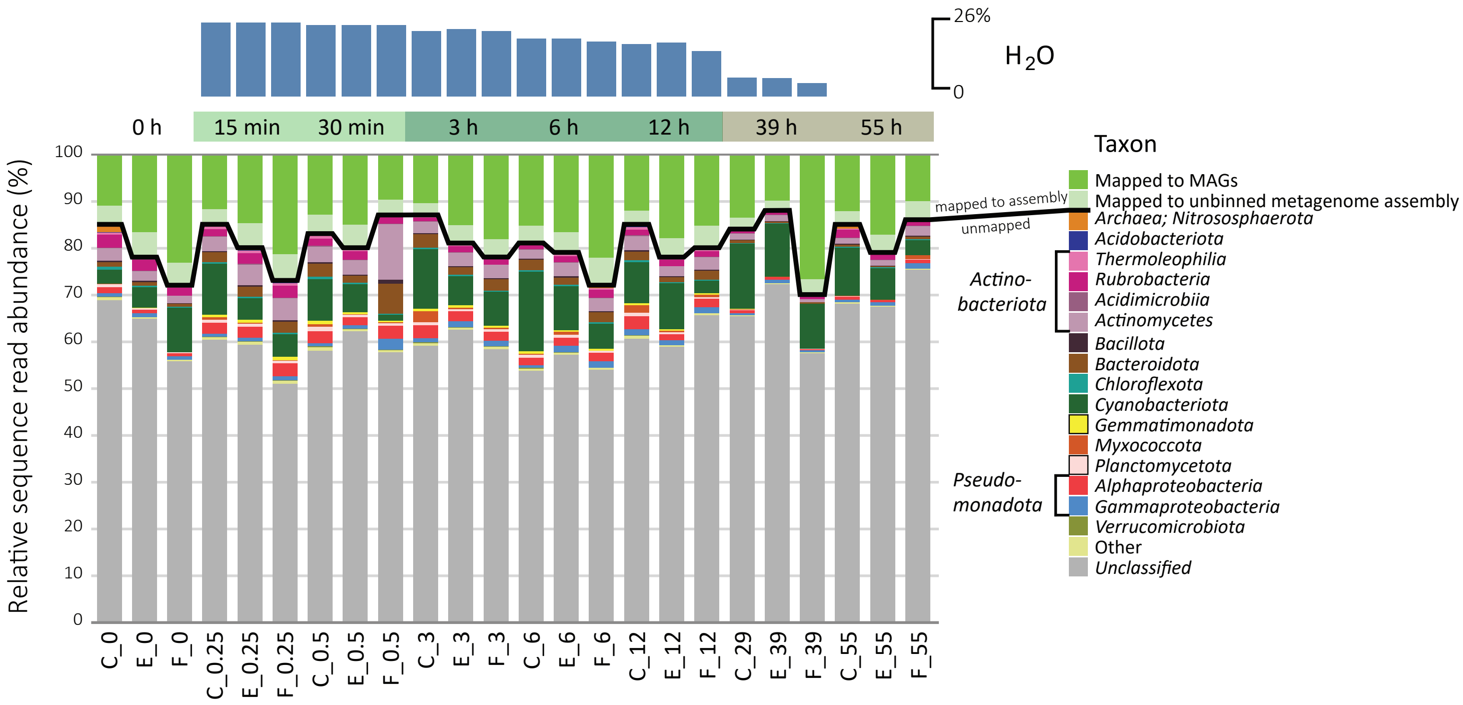

Supplementary Fig. 12. Classification and attribution of total mRNA reads in each sample.
On top of the bar chart is the fraction of reads that could be mapped to the metagenome assembly with an identity cut-off of 95%. Below the black line is the read fraction that could not be mapped to the assembly. The unmapped fraction was classified with kaiju (v. 1.9.1). Note that the taxa detected in the unmapped reads are largely the same taxa as represented by the MAGs (Supplementary Fig. 4). The unclassified reads had no hits to any sequence in the NCBI Refseq-Nr. 218 database.

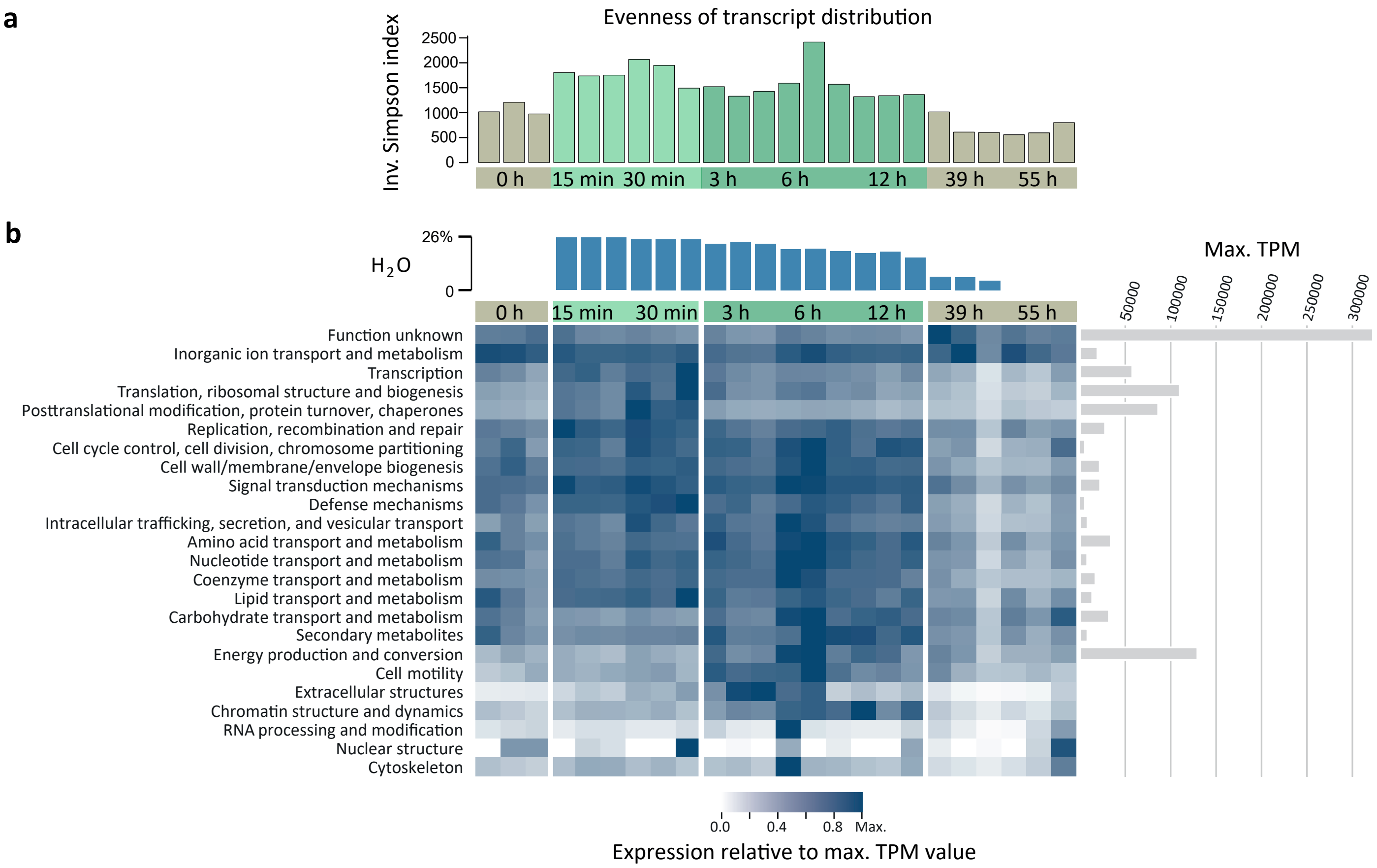

Supplementary Fig. 13. General transcription patterns across the time series.

a, Inverse Simpson index indicating evenness of transcript distribution among the different genes in the entire dataset. Smaller evenness means few genes receive high proportions of transcripts. Higher evenness indicates that more genes are expressed at more equal levels. **b,** Relative expression of COG functional categories. Genes were assigned to COG functional categories based on EggNOG mapper annotation of the metagenomes, described in Meier *et al.* (2021).

Supplementary Table 1. Precipitation at Sede-Boqer (Israel) weather station between 2015 and 2021.

Rain season	2015/16	2016/17	2017/18	2018/19	2019/20	2020/21
Precipitation (mm)	101.1	56.3	71.4	87.9	145.3	25.3
Days with precipitation	40	22	33	27	45	15
1-day precipitation (counts)	12	8	11	10	12	3
2-days precipitation (counts)	8	7	2	7	5	1
3-days precipitation (counts)	1	0	2	1	6	2
4-days precipitation counts	0	0	0	0	0	1
5-days precipitation (counts)	2	0	1	0	1	0
6-days precipitation (counts)	0	0	0	0	0	0
7-days precipitation (counts)	0	0	1	0	0	0

Source: Raw data provided by the meteorological observation site in Sede-Boqer (30.85972 N, 34.77944 E, 480 m.a.s.l)

Supplementary Table 2. Sequencing statistics of metatranscriptome libraries including samples water content.

Sample	Water content (in %)	Raw read pairs	Error corrected read pairs	rRNA reads	mRNA read pairs	Mapped to metagenome
C_0	< 1.2	66,268,397	63,960,267	9%	57,893,326	15%
E_0	< 1.2	42,484,756	40,198,680	16%	33,665,703	22%
F_0	< 1.2	43,047,033	40,146,649	8%	36,992,091	28%
C_0.25	26	63,819,485	61,893,037	24%	46,828,882	15%
E_0.25	26	57,022,890	54,901,647	10%	49,397,882	20%
F_0.25	26	57,983,409	56,094,666	18%	45,968,755	27%
C_0.5	n.d.	79,349,277	77,136,590	6%	72,239,930	17%
E_0.5	n.d.	42,663,084	40,205,934	10%	36,089,377	20%
F_0.5	n.d.	46,646,453	44,107,575	6%	41,381,228	13%
C_3	22.7	96,404,040	92,407,786	24%	70,547,093	13%
E_3	23.5	48,794,446	45,660,349	9%	41,424,597	19%
F_3	22.7	47,079,094	43,749,549	7%	40,589,102	22%
C_6	20.1	79,291,421	76,957,788	8%	70,547,093	19%
E_6	20.1	45,488,204	42,595,894	5%	40,331,765	21%
F_6	19.1	45,960,650	42,995,439	8%	39,767,851	28%
C_12	18.2	80,957,080	78,396,260	7%	72,627,149	15%
E_12	18.8	41,845,142	39,532,307	9%	36,023,190	22%
F_12	15.9	45,802,945	42,905,227	9%	38,904,335	20%
C_29	6.5	83,527,918	81,346,779	20%	64,803,326	16%
E_39	6.3	57,074,128	53,858,842	5%	51,108,010	12%
F_39	4.6	59,697,719	56,385,707	4%	54,334,481	30%
C_55	< 1.2	82,661,040	81,265,848	11%	72,463,255	15%
E_55	< 1.2	60,643,502	55,992,290	9%	50,881,195	21%
F_55	< 1.2	59,921,886	55,091,846	10%	49,784,680	14%

Supplementary Table 3. Technical data of tested materials in H_2 oxidation measurements.

**Description**	**Material/Treatment**	**Manufacturer**	**Catalogue No.**
20 mm stoppers	Butyl rubber N20	Glasgerätebau OCHS Laborfachhandel e.K.	102049
20 mm, clear silicone straight wall stoppers	Silicone	Supelco/Sigma-Aldrich (Vienna, Austria)	27235-U
Butyl rubber septa	Butyl rubber	IVA Analysen Technik (Meerbusch, Germany)	IVAVW101
Silicone septa*	Silicone	IVA Analysen Technik (Meerbusch, Germany)	IVA70212803

*As the thickness of these silicon septa was only 1.5 mm, the manufacturer recommended to place a butyl rubber septum in between the silicone septum and cap to ensure a gas-tight seal.

Supplementary Table 4. Minimal biomass generation rates determinable by NanoSIMS under the experimental conditions of this study. The displayed times indicate the maximal time that individual cells may take in replication (sole growth) or regeneration (sole maintenance) for being detected as active.

Incubation time [hours]	Min. detectable biomass generation rate [1/day]		Max. time for cellular replication [days]		Max. time for cellular regeneration [days]	
	obligate heterotroph	(chemo)autotroph	obligate heterotroph	(chemo)autotroph	obligate heterotroph	(chemo)autotroph
3 h	0.0291	0.0091	23.8	76.5	34.4	110.3
6 h	0.0145	0.0045	47.7	152.9	68.8	220.6
12 h	0.0073	0.0023	95.3	305.9	137.6	441.3
24 h	0.0036	0.0011	190.7	611.8	275.1	882.6

References

[1] Polerecky, L. *et al*. Calculation and interpretation of substrate assimilation rates in microbial cells based on isotopic composition data obtained by nanoSIMS. *Frontiers in Microbiology*, 12:621634 (2021).

[2] Kopf, S.H. *et al.* Heavy water and ^{15}N labeling with NanoSIMS analysis reveals growth-rate dependent metabolic heterogeneity in chemostats. *Environmental Microbiology*, 17:2542–2556 (2015).

[3] Kopf, S.H. *et al.* Trace incorporation of heavy water reveals slow and heterogeneous pathogen growth rates in cystic fibrosis sputum. *Proceedings of the National Academy of Sciences*, 113:E110–E116 (2016).

[4] Berry, D. *et al.*, Tracking heavy water (D_2O) incorporation for identifying and sorting active microbial cells. *Proceedings of the National Academy of Sciences*, 112:E194–E203 (2015).

[5] Sessions, A.L. *et al.* Calculation of hydrogen isotopic fractionations in biogeochemical systems. *Geochimica et Cosmochimica Acta*, 69:593–597 (2005).

[6] Wijker, R.S. *et al.* $^{2}H/^{1}H$ variation in microbial lipids is controlled by NADPH metabolism. *Proceedings of the National Academy of Sciences*, 116:12173–12182 (2019).

[7] Caro, T. A. *et al.* Hydrogen stable isotope probing of lipids demonstrates slow rates of microbial growth in soil. *Proceedings of the National Academy of Sciences* 120, e2211625120 (2023)

[8] Zhang, X. *et al*. Large D/H variations in bacterial lipids reflect central metabolic pathways. *Proceedings of the National Academy of Sciences*, 106:12580–12586 (2009).

[9] Baran, R. *et al.* Extensive turnover of compatible solutes in cyanobacteria revealed by deuterium oxide (D_2O) stable isotope probing. *ACS chemical biology*, 12:674–681 (2017).

[10] De Laeter, J.R. *et al.* Atomic weights of the elements. Review 2000 (IUPAC Technical Report). *Pure and applied chemistry*, 75:683–800 (2003).

[11] Wegener, G. *et al.* Assessing sub-seafloor microbial activity by combined stable isotope probing with deuterated water and ^{13}C-bicarbonate. *Environmental Microbiology*, 14:1517–1527 (2012).

[12] Wegener, G. *et al*. Tracking activity and function of microorganisms by stable isotope probing of membrane lipids. *Current Opinion in Biotechnology*, 41:43–52 (2016).

[13] Musat, N. *et al.* The effect of FISH and CARD-FISH on the isotopic composition of ^{13}C-and ^{15}N-labeled Pseudomonas putida cells measured by nanoSIMS. *Systematic and Applied Microbiology*, 37:267–276 (2014).

[14] Woebken, D. *et al.* Revisiting N_2 fixation in Guerrero Negro intertidal microbial mats with a functional single-cell approach." *The ISME Journal*, 9:485–496 (2015).

[15] Musat, N. *et al.* Tracking microbial interactions with NanoSIMS. *Current Opinion in Biotechnology*, 41:114–121 (2016).

[16] Stryhanyuk, H. *et al*. Calculation of single cell assimilation rates from SIP-NanoSIMS-derived isotope ratios: a comprehensive approach. *Frontiers in Microbiology*, 9:2342 (2018).

[17] Meyer, N.R. *et al.* NanoSIMS sample preparation decreases isotope enrichment: magnitude, variability and implications for single-cell rates of microbial activity. *Environmental Microbiology*, 23:81–98 (2021).

[18] Madigan, M.T. *et al.* in *Brock biology of microorganisms*. Vol. 15. Pearson Prentice Hall Upper Saddle River, NJ:, (2015).

[19] Kiernan, J. in *Histological and histochemical methods.* 5th edition, Scion Publishing Ltd, (2015)

[20] Fischer, C.R. *et al.* Stable-Isotope Probing reveals that hydrogen isotope fractionation in proteins and lipids in a microbial community are different and species-specific. *ACS Chemical Biology*, 8:1755–1763 (2013).

[21] Menzel, P., Ng, K. & Krogh, A. Fast and sensitive taxonomic classification for metagenomics with Kaiju. *Nat Communications* 7, 11257 (2016).

[22] Tamames, J., Cobo-Simón, M. & Puente-Sánchez, F. Assessing the performance of different approaches for functional and taxonomic annotation of metagenomes. *BMC Genomics* 20, 960 (2019).

[23] Meier, D. V. *et al*. Distribution of mixotrophy and desiccation survival mechanisms across microbial genomes in an arid biological soil crust community. *Msystems*, 6(1), 10-1128 (2021).

[24] Nauer, P.A. *et al.* Technical note: Inexpensive modification of exetainers for the reliable storage of trace-level hydrogen and carbon monoxide gas samples. *Biogeosciences*, 18:729–737 (2021).
