# Supplementary material for: Survival and rapid resuscitation permit limited productivity in desert microbial communities
Source: Nat Commun. 2024 Apr 17;15:3056. doi: 10.1038/s41467-024-46920-6 (PMC11519504; doi:10.1038/s41467-024-46920-6)
Supplement: Supplementary file 3 — Description of Additional Supplementary Files [file 41467_2024_46920_MOESM3_ESM.pdf]

Survival and rapid resuscitation permit limited productivity in desert microbial communities

Stefanie Imminger, Dimitri V. Meier, Arno Schintlmeister, Anton Legin, Jörg Schnecker,

Andreas Richter, Osnat Gillor, Stephanie A. Eichorst, Dagmar Woebken

Description of Additional Supplementary Files

File Name: **Supplementary Data 1**
Description: A Microsoft Excel file summarizing the imaged regions of interest (ROIs) representing individual cells, their size, the measurement uncertainty (Poisson error) and isotopic content both of individual single cells and cyanobacterial filaments and the corresponding controls at different measurement time point.

File Name: **Supplementary Data 2**
Description: A Microsoft Excel file summarizing the numbers of genes detected as expressed for each MAG in each sample as well as numbers of genes differentially expressed either between subsequent time points or between experimental phases.

File Name: **Supplementary Data 3**
Description: A Microsoft Excel file containing detailed functional annotations as well as per-MAG-normalized expression values (in transcripts per million) and the results of DeSeq2 analyses (Log2 fold change and adjusted *p*-values) for each gene analyzed for this study.

File Name: **Supplementary Data 4**
Description: A Microsoft Excel file containing the catalogue numbers of all chemicals used in this study
