# Supplementary material for: Survival and rapid resuscitation permit limited productivity in desert microbial communities
Source: Nat Commun. 2024 Apr 17;15:3056. doi: 10.1038/s41467-024-46920-6 (PMC11519504; doi:10.1038/s41467-024-46920-6)
Supplement: Supplementary file 8 — Reporting Summary [file 41467_2024_46920_MOESM8_ESM.pdf]

nature portfolio

Corresponding author(s): Dagmar Woebken

Last updated by author(s): Feb 25, 2024

Reporting Summary

Nature Portfolio wishes to improve the reproducibility of the work that we publish. This form provides structure for consistency and transparency in reporting. For further information on Nature Portfolio policies, see our Editorial Policies and the Editorial Policy Checklist.

Statistics

For all statistical analyses, confirm that the following items are present in the figure legend, table legend, main text, or Methods section.

n/a	Confirmed	
☐	☒	The exact sample size (*n*) for each experimental group/condition, given as a discrete number and unit of measurement
☐	☒	A statement on whether measurements were taken from distinct samples or whether the same sample was measured repeatedly
☐	☒	The statistical test(s) used AND whether they are one- or two-sided<br>*Only common tests should be described solely by name; describe more complex techniques in the Methods section.*
☐	☒	A description of all covariates tested
☐	☒	A description of any assumptions or corrections, such as tests of normality and adjustment for multiple comparisons
☐	☒	A full description of the statistical parameters including central tendency (e.g. means) or other basic estimates (e.g. regression coefficient) AND variation (e.g. standard deviation) or associated estimates of uncertainty (e.g. confidence intervals)
☐	☒	For null hypothesis testing, the test statistic (e.g. *F*, *t*, *r*) with confidence intervals, effect sizes, degrees of freedom and *P* value noted<br>*Give P values as exact values whenever suitable.*
☒	☐	For Bayesian analysis, information on the choice of priors and Markov chain Monte Carlo settings
☒	☐	For hierarchical and complex designs, identification of the appropriate level for tests and full reporting of outcomes
☒	☐	Estimates of effect sizes (e.g. Cohen's *d*, Pearson's *r*), indicating how they were calculated

Our web collection on statistics for biologists contains articles on many of the points above.

Software and code

Policy information about availability of computer code

Data collection	rRNA-depleted RNA-seq libraries were sequenced on Illumina HiSeq 2500 in 2x125 bp mode, and an average read depth of 60 +- 16 mio read pairs.<br>Sequence reads were trimmed using BBduk v.37.61 with default parameters and error-corrected using Bayes-Hammer module of SPAdes assembler v.3.11. All reads resembling ribosomal RNA were removed from the data set by mapping the reads to the SILVA SSU132 and LSU132 and the 5S rRNA database with a sequences identity of >70% using BBmap v.37.61. The remaining reads were mapped to previously published metagenome contigs with an identity and post-mapping identity of 99% and 97%, respectively. In addition, only pairs where both reads map in the correct orientation and correct insert size were accepted. The numbers of error-corrected and mapped reads are summarized in Supplementary Table 2.<br>In order to assign mapped reads to gene calls, the bam BAM files generated by BBmap were processed with featureCounts from the Subread package v. 2.0.0 resulting in a read count per gene per sample table, which was used for downstream processing in R v. 3.6.1.
Data analysis	Statistical analysis of gene expression was performed in R v- 3.6.1 using the vegan v. 2.5.4 and DESeq2 v. 1.26.0 packages. Searching of genes by functional annotation in the entire dataset was done by SQL queries in SQLite browser v. 3.11.2. Various simple data table transformations and plotting of transcription heat maps was done in python using the pandas v. 1.3.3 and seaborn v. 0.11.1 modules. .<br>The analysis is documented in code deposited on GitHub (https://doi.org/10.5281/zenodo.10657361).

For manuscripts utilizing custom algorithms or software that are central to the research but not yet described in published literature, software must be made available to editors and reviewers. We strongly encourage code deposition in a community repository (e.g. GitHub). See the Nature Portfolio guidelines for submitting code & software for further information.

Data

Policy information about availability of data

All manuscripts must include a data availability statement. This statement should provide the following information, where applicable:

- Accession codes, unique identifiers, or web links for publicly available datasets
- A description of any restrictions on data availability
- For clinical datasets or third party data, please ensure that the statement adheres to our policy

The quality-filtered mRNA reads have been uploaded to the European Nucleotide Archive under the project number PRJEB52014 (https://www.ebi.ac.uk/ena/browser/view/PRJEB52014). The nanoSIMS data generated in this study are provided in the Supplementary Data 1. Data used to generate the figures in this manuscript are summarized in the Source Data File_Figures.
The previously generated metagenome assembly and metagenome-assembled genomes (Meier et al. 2021) that were used as a reference for transcript mapping are available through public DNA sequence archives under project number PRJEB36534 (https://www.ebi.ac.uk/ena/browser/view/PRJEB36534). Source data for presented figures are provided with this paper as a Source Data file. The following rRNA databases were used for filtering out the remaining rRNA reads from the transcriptomes: SILVA SSU132 (https://www.arb-silva.de/fileadmin/silva_databases/release_132/Exports/SILVA_132_SSURef_Nr99_tax_silva.fasta.gz), SILVA LSU132 (https://www.arb-silva.de/fileadmin/silva_databases/release_132/Exports/SILVA_132_LSURef_tax_silva.fasta.gz), 5S rRNA database (http://combio.pl/rrna/download/).

Research involving human participants, their data, or biological material

Policy information about studies with human participants or human data. See also policy information about sex, gender (identity/presentation), and sexual orientation and race, ethnicity and racism.

Reporting on sex and gender	This study does not involve human participants or human data.
Reporting on race, ethnicity, or other socially relevant groupings	This study does not involve human participants or human data.
Population characteristics	This study does not involve human participants or human data.
Recruitment	This study does not involve human participants or human data.
Ethics oversight	This study does not involve human participants or human data.

Note that full information on the approval of the study protocol must also be provided in the manuscript.

Field-specific reporting

Please select the one below that is the best fit for your research. If you are not sure, read the appropriate sections before making your selection.

☒ Life sciences ☐ Behavioural & social sciences ☐ Ecological, evolutionary & environmental sciences

For a reference copy of the document with all sections, see nature.com/documents/nr-reporting-summary-flat.pdf

Life sciences study design

All studies must disclose on these points even when the disclosure is negative.

Sample size	Sampling time points for metatranscriptomics were chosen to cover different intervals of a hydration desiccation curve: i) Reference samples before the start and upon completion of the experiment (dry undisturbed crusts and dry crust after the hydration and desiccation). ii) More frequent sampling directly after hydration where most change in transcription was expected, less frequent sampling in the phase where temperature, light and hydration conditions were stable. The number of metatranscriptomic replicates per time point (n=3) was chosen based on current community standards in environmental transcriptomics. No statistical methods were applied to estimate the necessary replicate number.<br><br>For isotopic enrichment analysis via NanoSIMS, 211-248 individual cells and 6-7 cyanobacterial filaments per time point were measured. The sample size was not determined by statistical method but was according to current community standards in NanoSIMS analysis of environmental samples. To confirm our observed activity pattern, a time series experiment including 4 samples was applied.<br><br>We observed congruent patterns in our H2 consumption assay across 3 replicates, therefore concluded this was sufficient for the data/pattern we report in this paper.
Data exclusions	No data were excluded
Replication	Biological replication for the metatranscriptomics experiment is provided by the fact that three different pieces of soil crust were harvested at each of the 8 time points. Meaning that the experiment consisted of 24 (3x8) separate incubations stopped at different time points. This replication was successful, however, the entire experiment was performed only once.

	Individual cells in the NanoSIMS investigation represent biological replicates.<br>H2 consumption assay were successfully performed in 3 replicates.<br>Similar patterns across replicates confirm successful replication.
Randomization	The replicate samples were taken from three random patches of cyanobacterial soil crust in a distance of >20 m to each other.<br>A pre-screening was performed to ensure that same organisms are present in all soil crusts to allow for a correct comparison and replication.
Blinding	The harvesting of soil crust at different time points of the rehydration time series was done blindly. No pre-selection of which crust pieces to sample at which time point was done.

Reporting for specific materials, systems and methods

We require information from authors about some types of materials, experimental systems and methods used in many studies. Here, indicate whether each material, system or method listed is relevant to your study. If you are not sure if a list item applies to your research, read the appropriate section before selecting a response.

Materials & experimental systems

n/a	Involved in the study
☒	☐ Antibodies
☒	☐ Eukaryotic cell lines
☒	☐ Palaeontology and archaeology
☒	☐ Animals and other organisms
☒	☐ Clinical data
☒	☐ Dual use research of concern
☒	☐ Plants

Methods

n/a	Involved in the study
☒	☐ ChIP-seq
☒	☐ Flow cytometry
☒	☐ MRI-based neuroimaging
